# Dynamic Morphological Transformation and Self‐Assembly of DNA‐Functionalized Cellulose Nanocrystal Building Blocks

**DOI:** 10.1002/cssc.202500341

**Published:** 2025-06-17

**Authors:** Jinsu Park, Youngeun Kim, Seung‐Yeop Kwak

**Affiliations:** ^1^ Department of Materials Science and Engineering Seoul National University 1 Gwanak‐ro Gwanak‐gu, Seoul 08826 South Korea; ^2^ Research Institute of Advanced Materials (RIAM) Seoul National University 1 Gwanak‐ro Gwanak‐gu, Seoul 08826 South Korea; ^3^ Institute of Engineering Research Seoul National University 1 Gwanak‐ro Gwanak‐gu,Seoul 08826 South Korea

**Keywords:** artificial cellulose, cellulose nanocrystal, CNC‐DNA hybrids, DNA‐mediated self‐assembly, microscale polysaccharidal assembly architecture

## Abstract

Self‐assembly of cellulose nanocrystals represents one of important pillars of nanoscience that integrates natural design motifs into development for sustainable solutions in key industries. However, there is only a limited number of methods that confer manipulation of interparticle interaction between cellulose nanocrystal building blocks and synthesis of well‐defined self‐assembly architectures. Herein, a DNA‐mediated strategy that enables a dynamic stepwise rod‐to‐sphere‐to‐rod morphological transformation of cellulose building blocks is introduced, culminating in the formation of slab‐like cellulose architectures in colloidal states. This work establishes a strategic bridge between cellulose nanocrystal assembly and programmable anisotropic nanoparticle systems while addressing a long‐standing challenge in DNA nanotechnology to producing scalable, biocompatible micro‐scale self‐assembly architectures. This work is envisioned that it may galvanize further research that accelerate the development of transformative solutions to address unmet challenges in medicine, energy, and soft robotics, particularly as carriers and scaffolds.

## Graphical abstract

We present a simple strategy for dynamic rod‐to‐sphere‐to‐rod morphological transformation and DNA‐mediated self‐assembly of cellulose nanocrystal building blocks into slab‐like architectures in colloidal states. Our approach opens a sustainable, scalable pathway toward biocompatible self‐assembly architectures that may address critical challenges in transformative applications such as medicine, energy, and soft robotics.

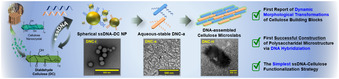

## 1 Introduction

Self‐assembly of anisotropic nanoparticles (NPs) or building blocks (BBs) like rods, prisms, and cubes have long inspired researchers across multiple disciplines for their asymmetric geometry and directional interactions.^[^ ^1^, ^2^, ^3^, ^4^ ^]^ The tunability of interparticle interactions by incorporating various binding forces (e.g., DNA hybridization, hydrogen bonding, Van der Waals, and electrostatic) enables realization of convergent materials properties (e.g., electrical, optical, mechanical, plasmonic, magnetic, and biological) beyond those of individual BBs that are desirable for advanced applications.^[^ ^1^, ^2^, ^3^, ^4^, ^5^ ^]^

This phenomenon is deeply rooted in the intricate micro‐/macrostructures found in nature like wood, which achieve anisotropic architecture, light weight, and thermal stability through the assembly of fibrillar cellulose building blocks.^[^ ^6^, ^7^, ^8^ ^]^ Cellulose nanocrystals (CNC, $C_{6}H_{10}O_{8}$)–extracted from these natural sources–are intrinsically rod‐like anisotropic NP BBs (3–50 nm wide; 100–200 nm long) that harness bioabundancy, mechanical robustness, cost‐efficiency, scalability, biodegradability, and biocompatibility.^[^ ^9^, ^10^, ^11^, ^12^, ^13^ ^]^ Especially, their self‐assembly into chiral nematic order give rise to macroscopic iridescent optical properties that have been extensively studied and exploited for photonic colorants, security features, sensors, and coatings and so on.^[^ ^9^, ^10^, ^11^, ^12^, ^13^, ^14^, ^15^, ^16^, ^17^, ^18^, ^19^ ^]^ Moreover, self‐assembly of CNCs functionalized with chemical moieties or graft polymers by accessing their surface chemistry (e.g., surface hydroxyl groups (—OH), reducing end‐group aldehydes (REG R—CHO)) exhibited different structure‐dependent behavior and collective properties with promising applications as sustainable solutions in key industries.^[^ ^10^, ^11^, ^12^, ^14^, ^16^, ^19^ ^]^

Despite the potential of CNC self‐assembly structures in a wide range of advanced applications,^[^ ^20^ ^]^ artificial methods that afford their construction and structural control are often limited to evaporation‐induced self‐assembly that yields solid‐state CNC films.^[^ ^9^, ^11^, ^13^, ^14^, ^19^ ^]^ Compared to those devised to assemble inorganic or supramolecular BBs, bottom‐up manipulation over interparticle interaction and arrangements of CNC BBs remain challenging due to nonuniform size distribution and functionalization of surface chemistry.^[^ ^7^, ^18^ ^]^ Given how the integration of programmability into the self‐assembly of inorganic or polymeric NPs has ushered in new era of nanoscience,^[^ ^1^, ^21^, ^22^, ^23^, ^24^ ^]^ developing an efficient method to prepare and control cellulose self‐assembly architectures may not only unveil nature's design principles for functional materials but also advance sustainable engineering solutions.

In our previous studies, we demonstrated that CNC NPs can be assembled into a long‐range ordered constructs^[^ ^25^ ^]^ and dendrite‐like microstructures^[^ ^26^ ^]^ under sufficient driving forces, such as alkene crosslinking via frontal ring‐opening metathesis reaction and H—F bond‐mediated fluoropolymer adsorption, respectively. Based on these findings, we hypothesized that the more delicate control of CNC interparticle arrangement may be accessed through DNA hybridization via specific base‐paring interactions (A—T, G—C).^[^ ^27^ ^]^ Indeed, a number of studies have functionalized CNC BBs with single‐stranded DNAs (ssDNAs) to CNC BBs using azide‐alkyne cycloaddition,^[^ ^27^ ^]^ carbodiimide coupling,^[^ ^28^ ^]^ and electrostatic interaction,^[^ ^29^ ^]^ to induce DNA‐mediated CNC self‐assembly. However, construction of CNC BB architectures with distinct morphological characteristics are yet to be reported.

**Figure 1:**
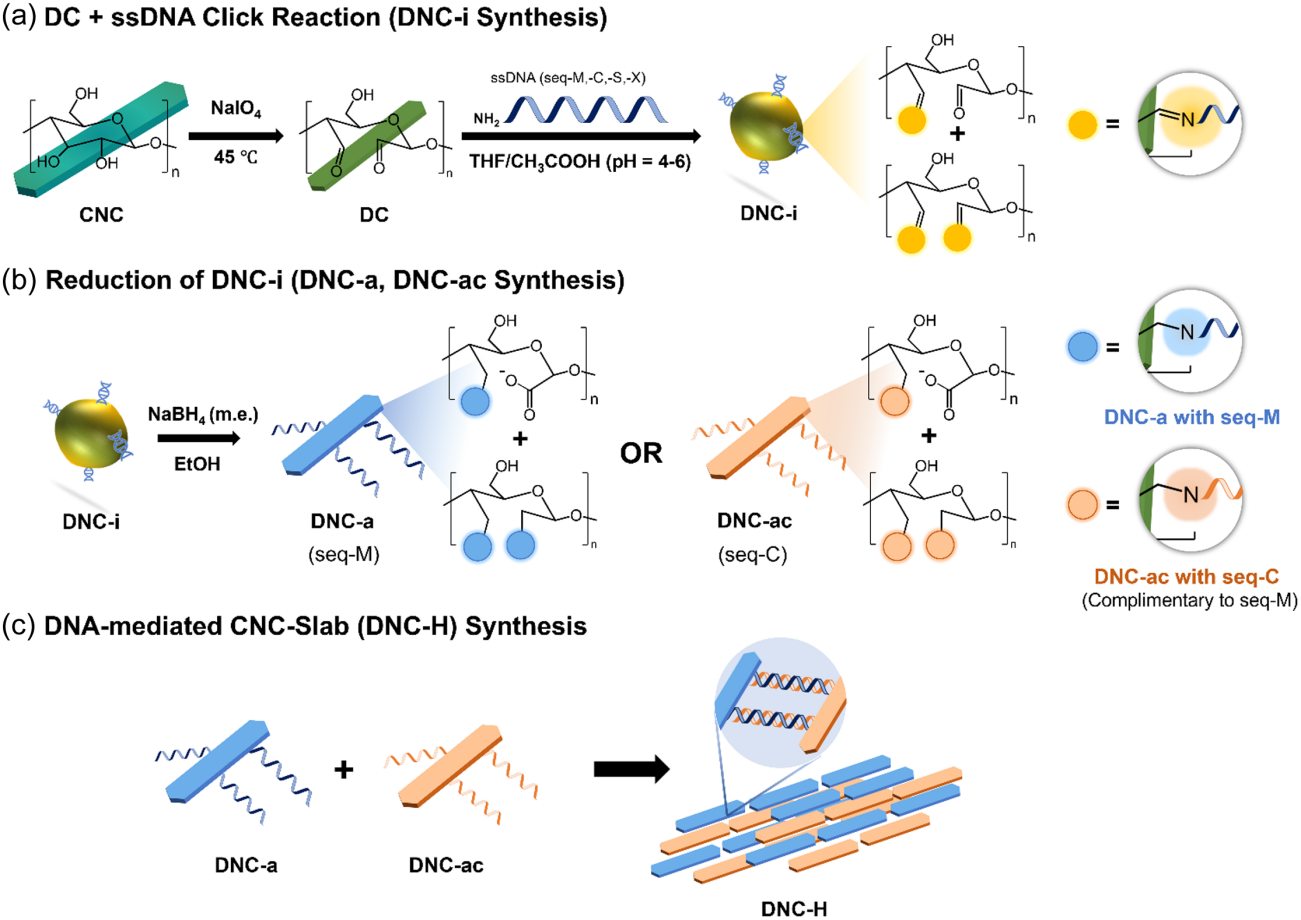
a) A schematic showing the reaction conditions of ssDNA‐functionalization of dialdehyde cellulose (DNC‐i). b) The reduction reaction of DNC‐i was performed in ethanol, and in excess of reducing agent (DNC‐a, DNC‐ac). Then, c) slab‐like cellulose microstructures were produced by mixing two batches of complementary DNC‐a building blocks in water.

Herein, we demonstrate an efficient DNA‐mediated strategy that enable dynamic morphological transformation and self‐assembly into microscale slab‐like architectures of cellulose building blocks. First, we observed a dynamic rod‐to‐sphere‐to‐rod structural transition in CNC BBs upon click reaction‐mediated ssDNA functionalization and subsequent reduction of ssDNA‐cellulose linkage. Then, microscale slab‐like cellulose architecture was produced in colloidal state via DNA hybridization (**Figure** **1** and S1, Supporting Information). To the best of our knowledge, this study provides the first demonstration of dynamic morphological control and arrangement of cellulose BBs into defined structures in a colloidal state. This work establishes a strategic pathway for designing cellulose self‐assembly and integrating integration into anisotropic NP self‐assembly nanotechnology, bridging the gap between bio‐derived and precisely engineered anisotropic BBs. From a DNA nanotechnology perspective, our method addresses a long‐standing challenge in the field to achieve efficient, scalable assembly of organic NPs into micro‐scale architectures, without reliance on complex surface modification strategies or templating agents. This class of bioderived BBs and micro‐assembly architectures, offering intrinsic anisotropy, ease of chemical modification, biocompatibility, and cost‐efficiency, holds promise for transformative application in medicine, energy, and soft robotics as carriers and scaffolds.

## 2 Results and Discussion

Similarly to the challenges faced in functionalizing inorganic NP BBs, one of the main hurdles in functionalizing CNCs with ssDNA lies in simplifying the grafting procedure while maximize the its efficacy.^[^ ^27^ ^]^ Previous approaches to address these challenges involved surface modifications of CNCs via bromination,^[^ ^27^ ^]^ carboxylation,^[^ ^28^ ^]^ and epoxidation^[^ ^29^ ^]^ of their surface –OH groups.

**Figure 2:**
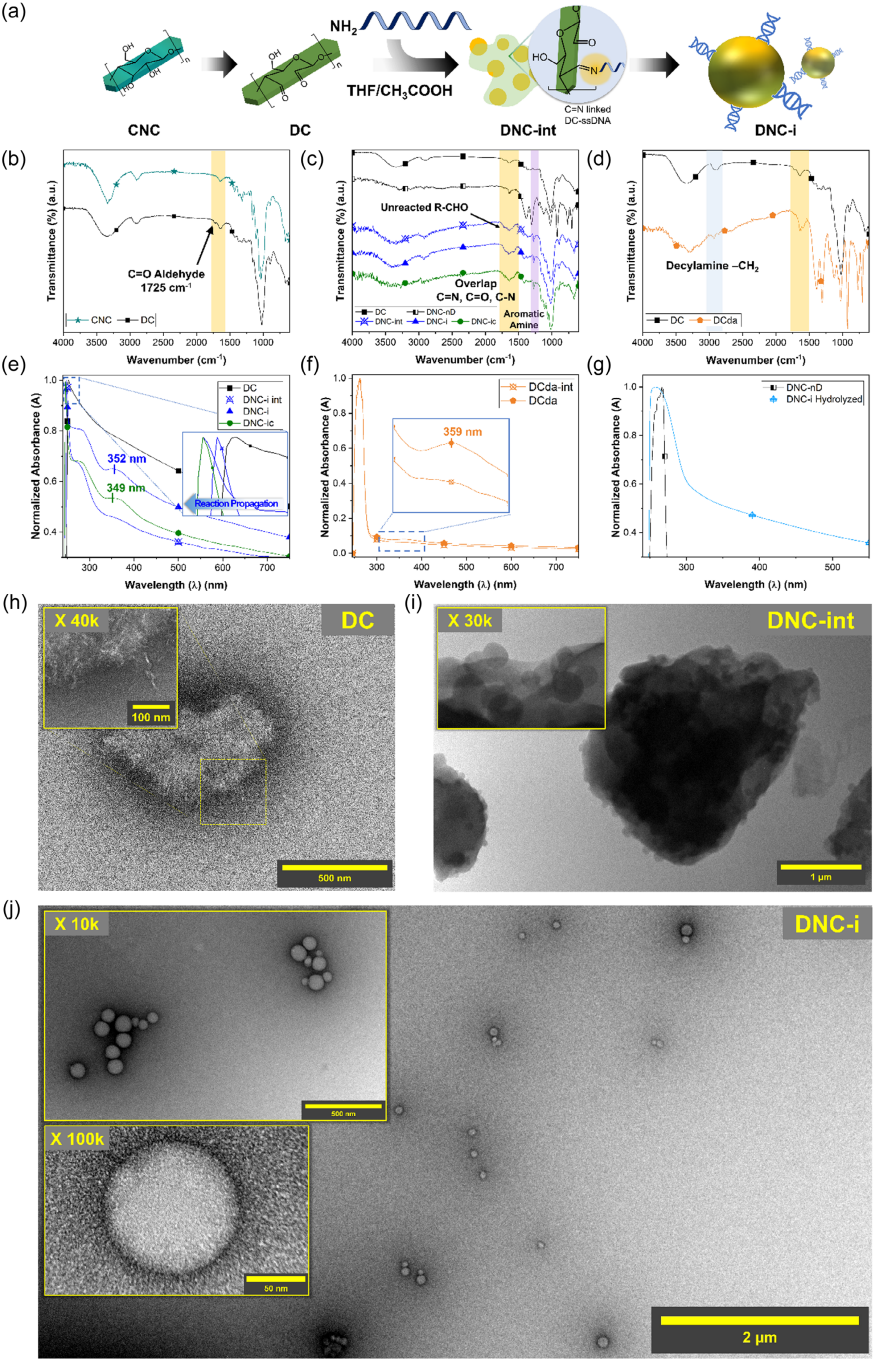
a) A schematic showing the processes of converting CNCs to DCs, and subsequent formation of spherical DNC‐i/‐ic NPs in THF/CH_3_COOH by facilitating the formation of C=N linkages. The ATR‐FTIR spectra of b) CNC, DC, c) DNC‐int, DNC‐i, DNC‐ic, DNC‐nD, and d) DCda confirmed the formation of C=N bonds and the presence of ssDNA in DNCs. e) The UV‐vis spectra of these specimens further confirmed the gradual formation of imine bonds at ≈350 nm. This peak assignment was validated through f) IR spectra of DCda, and g) the its disappearance after imine‐hydrolysis in water. The TEM images of h) DC, i) DNC‐int, and j) DNC‐i revealed that spherical NPs were produced from the ssDNA‐functionalization of DCs.

In this work, we hypothesized that dialdehyde cellulose (DC), compared to surface‐modified CNCs, would facilitate simpler and more effective reaction with amine‐functionalized ssDNA due to the click chemistry between R—CHO and amines (Figure 1a, S1a, Supporting Information). DCs are derived from CNCs through periodate oxidation, which selectively cleaves the carbon–carbon bonds at the anhydroglucose unit (AGU) C2, C3 positions, converting their –OH groups into R—CHO groups.^[^ ^30^ ^]^ The conversion of CNC to DC was confirmed via attenuated total reflectance Fourier‐transform infrared (ATR‐FTIR) spectroscopy, which revealed the R—CHO signal at 1725 cm^−1^ (**Figure** **2**a, b). Here, the –OH stretching signals from water absorbed by CNC and DC were observed at 1640 cm^−1^.^[^ ^30^ ^]^ Additionally, the zeta potential (*ζ*) analysis indicated a surface charge of $\zeta_{\text{CNC}}$ = −48.3 ± 2.5 mV and $\zeta_{\text{DC}}$ = −27.3 ± 2.6 mV, reflecting the chemical transformation of CNCs to DCs.

The choice of solvent systems may impact the ssDNA grafting process onto DC because the solvent often influences cellulose modification pathways, reactant interactions, and the properties of the resulting products.^[^ ^26^ ^]^ Therefore, it is important to note that in our case, this solvent should prevent C=N bond hydrolysis, DC dissolution, and ssDNA aggregation. Water, dimethyl sulfoxide or dimethylformamide, and ethanol were therefore excluded due to these limitations. Consequently, tetrahydrofuran (THF), which are commercially used for DNA or peptide synthesis, was selected as the primary solvent. Additionally, acetic acid (${\text{CH}}_{3}\text{COOH}$ was added into (THF/${\text{CH}}_{3}\text{COOH}$ 25:1 w/w, pH ≈ 4–6) to facilitate a robust DC–ssDNA functionalization (Figure S1b, Supporting Information).

The formation of imine (C=N) linkages between DC and ssDNA in imine‐linked DC‐ssDNA (DNC‐i) was confirmed by several spectral and electrostatic changes. For instance, the R—CHO signal at 1725 cm^−1^ was consumed, while a broad peak appeared in the 1530–1754 cm^−1^ region which was attributed to the overlapping contributions from C=N (1650 cm^−1^), unreacted C=O (1710 cm^−1^), and C—N (1566 cm^−1^) signals (Figure 1a, 2a, c). A significant reduction in *ζ* value to $\zeta_{\text{DNC‐i}}$ = −44.6 ± 1.7 mV further supported the successful functionalization of ssDNA by C=N linkage. Especially, the IR R—CHO signal was retained in the intermediary product DNC‐int ($\zeta_{\text{DNC‐int}}$ = −32.0 ± 1.5 mV) due to insufficient imine‐formation and ssDNA‐functionalization. In contrast, control sample that was produced in the absence of ssDNA (DNC‐nD) showed neither these spectral features nor significant changes in *ζ* value ($\zeta_{\text{DNC‐nD}}$ = −25.9 ± 1.1 mV). When complementary ssDNA was introduced to DCs (DNC‐ic), similar spectral features in the 1530–1754 cm^−1^ region and *ζ* values were observed ($\zeta_{\text{DNC‐ic}}$ = −43.8 ± 0.9 mV) (Table S1, Supporting Information). To validate these findings further, a control group was prepared by substituting amine‐functionalized ssDNA with decylamine (DCda). DCda exhibited disappearance of the R—CHO signal and $\zeta_{\text{DCda}}$ = −31.1 ± 3.1 mV (Figure 2d, Table S1, Supporting Information). The presence of C=N bonds in DNC‐i, DNC‐ic, and DCda was confirmed via N1s profile of powder X‐ray photoelectron spectroscopy (XPS), which exhibited a peak at ≈400 eV (Figure S2, Supporting Information). Interestingly, the N1s XPS profiles of DNC‐i and DNC‐ic also revealed pyridinic C—N signals at around 398 eV, characteristic of ssDNA bases, which were absent in DCda. These findings were further corroborated by IR spectroscopy, which revealed sharp signals at 1277 cm^−1^ in DNC‐i and DNC‐ic, corresponding to aromatic/pyridinic C—N from nucleotide bases, but not observed in DCda (Figure 2c, d).^[^ ^31^, ^32^ ^]^ Moreover, a comparison of the IR spectra between neat ssDNA and DNC‐i clearly highlighted this characteristic peak at 1277 cm^−1^, confirming the presence of ssDNA in DNC‐i (Figure S3, Supporting Information). The dynamic transformation of the DC‐ssDNA structures through imine linkage was further characterized using UV‐visible (UV‐vis) spectroscopy. The ssDNA absorption signal exhibited a red‐shift from 262 to 284 nm upon conjugation to DC (Figure S4, Supporting Information, 2e). Moreover, a gradual increase in the signal intensity of a new peak at ≈350 nm was observed as the reaction progressed from DC to DNC‐i/‐ic but disappeared in water due to hydrolysis of imine bond in a protonated environment (Figure 2e, g). This phenomenon was also observed from DCda and its intermediary product (DCda‐int) at a similar wavelength (Figure 2e, f). Therefore, we deduced that the peak at 350 nm represented the C=N linkage.

Interestingly, we also observed a gradual blue‐shift of cellulose signal from 253 to 246 nm which suggested potential size reduction^[^ ^33^ ^]^ or aggregation^[^ ^34^ ^]^ of NPs (Figure 2a,e). The transmission electron microscopy (TEM) images of DC, DNC‐int, DNC‐i revealed a dynamic transformation of a rod‐like DCs into spherical DNC‐i NPs (Figure 2h–j). As compared to the typical cases of CNCs where well‐dispersed individual NPs were observed ($l_{\text{CNC}}$ = 143.1 ± 25.8 nm; $w_{\text{CNC}}$ = 12.7 ± 2.1 nm), DCs revealed aggregations of smaller rod‐like NPs ($l_{\text{DC}}$ = 44.3 ± 6.2 nm; $w_{\text{DC}}$ = 7.0 ± 1.7 nm) (Figure 2h, S5a, Supporting Information). Then, intermediate DNC‐int showed spherical NPs ($d_{\text{DNC‐int}}$ = 121 ± 30.1 nm) being nucleated from micron‐sized aggregates (Figure 2i, S5c, Supporting Information). After 24 h, spherical DNC‐i NPs ($d_{\text{DNC‐i}}$ = 91.1 ± 21.9 nm) were formed (Figure 2j, S5d, Supporting Information). Similar geometrical attributes were observed from DNC‐ic, which exhibited a slight increase in size ($d_{\text{DNC‐ic}}$ = 117.4 ± 22.9 nm) (Figure S4e, Supporting Information). This phenomenon, however, was not observed in DNC‐nD (Figure S5b, Supporting Information), while DCda formed irregularly shaped NPs ≈5 times larger than DNC‐i (Figure S6, Supporting Information). Based on these findings, we deduced that the formation of imine bonds between DCs and the amine sources may have driven the morphological transition from rod‐like to spherical NPs.

The effects of these morphological transformation on the crystal structures of DC and DNC derivatives were investigated via powder X‐ray diffraction (XRD) analysis. While most of the typical cellulose lattice planes were observed in DC: (1–1 0), (1 1 0), (0 1 2), (2 0 0), and (0 0 4) at around 2θ = 14.9°, 16.8°, 19.5°, 20.5°, 22.3°, 34.6°, respectively, a new peak emerged at ≈2θ = 18.3° (Figure S7a–f, Supporting Information). This peak was previously unassigned^[^ ^35^ ^]^ and likely corresponds to a cellulose lattice plane altered by AGU cleavage during periodate oxidation. Demonstratively, significant reductions in crystallinity indices (CrI) and crystallite sizes (*D*) were observed during the CNC‐to‐DC conversion (${\text{CrI}}_{\text{CNC}}$ = 87.0%, $D_{\text{CNC}}$ = 3.8 nm; ${\text{CrI}}_{\text{DC}}$ = 77.8%, $D_{\text{DC}}$ = 2.7 nm), which may attest to the changes induced to the crystal structures of cellulose BBs (Table S2, Supporting Information).^[^ ^30^ ^]^ During ssDNA‐functionalization, CrI initially decreased (${\text{CrI}}_{\text{DNC‐int}}$ = 61.9%), suggesting potential amorphization, but partially recovered upon spherical NP formation (${\text{CrI}}_{\text{DNC‐i}}$ = 77.0% and ${\text{CrI}}_{\text{DNC‐ic}}$ = 66.4 %), aligning with the nucleation or recrystallization process of spherical DNC‐i/‐ic NPs observed from the TEM analyses (Table S2, Supporting Information). We aimed to test our hypothesis by comparing the TEM images and XRD patterns of DCda. Recall that DCda revealed cores that exhibited mildly transparent, potentially suggesting reduced crystalline regions (Figure S6a–d). The XRD pattern of DCda exhibited significantly reduced diffraction intensity, correlating with their amorphous cores (Figure S6, S7f, Supporting Information). These findings collectively confirm that the formation of C=N bonds between DCs and amine‐functionalized ssDNA drives the morphological transformation from rod‐like DCs to spherical DNC‐i NPs, a process involving amorphization and crystallization of cellulose BBs.

**Figure 3:**
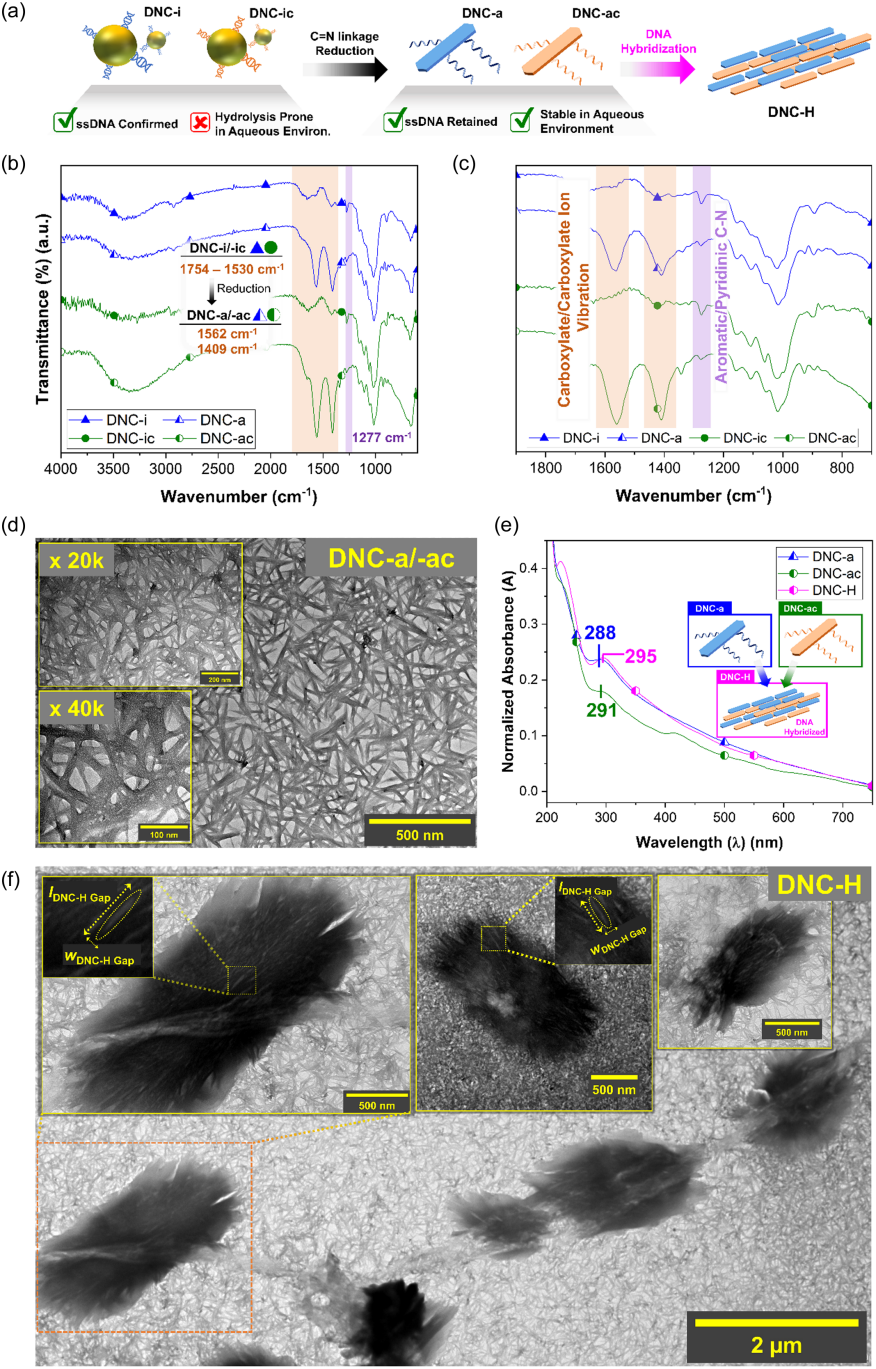
a) A schematic showing the processes involving reduction of DNC‐i/‐ic to DNC‐a/‐ac, and the construction of cellulose microstructure via DNA hybridization (DNC‐H). b–c) The ATR‐FTIR spectra of showing the spectral differences of reducing DNC‐i and DNC‐ic to DNC‐a and DNC‐ac. Especially, the peak at 1277 cm^−1^ showed preservation of ssDNAs. d) TEM images of DNC‐a and ‐ac. e) The UV‐vis spectra of DNC‐a, ‐ac, and ‐H showed DNA hybridization resulted in red‐shift of ssDNA peak. f) TEM images showing DNA‐mediated self‐assembled cellulose microstructures (DNC‐H).

Promoting DNA hybridization‐mediated self‐assembly between DNC‐i and DNC‐ic posed two primary challenges. Both DNC‐i and DNC‐ic were prone to hydrolysis in aqueous environment, reverting to their precursor DC forms ($l_{\text{DNC‐i,hydrolyzed}}$ = 38.6 ± 5.2 nm, $w_{\text{DNC‐i,hydrolyzed}}$ = 6.3 ± 1.0 nm) (Figure S8, Supporting Information). This phenomenon was also demonstrated by the disappearance of ssDNA signal in the 262–283 nm region due to the breakdown of the imine linkage between DC core and ssDNA (Figure 2g). However, DNA hybridization process, enabled by Watson‐Crick base pairing, necessitates water‐based solvent to stabilize the hydrogen bonds formed between nucleotide bases. To address these issues, DNC‐i and DNC‐ic were reduced to DNC‐a ($\zeta_{\text{DNC‐a}}$ = −17.7 ± 1.5 mV) and DNC‐ac ($\zeta_{\text{DNC‐ac}}$ = −17.6 ± 1.9 mV), respectively, to promote DNA‐mediated self‐assembly of cellulose BBs (Figure 1b, 1c, **3**a). The IR spectra of DNC‐a exhibited signals for asymmetrical and symmetrical stretching of carboxylate/carboxylate ion (COO^−^) at ≈1562 cm^−1^ and 1409 cm^−1^, respectively.^[^ ^36^ ^]^ Moreover, the aromatic/pyridinic C—N signal at 1277 cm^−1^ was preserved, indicating the retention of ssDNA after the reduction process (Figure 3b, c). On the contrary, in the control sample DCda‐a ($\zeta_{\text{DCda‐a}}$ = −6.2 ± 1.0 mV), the reduction product of DCda, a similar COO^−^ signals were observed, but the characteristic ssDNA peak at 1277 cm^−1^ was absent (Figure S9, Supporting Information). These findings confirmed that the reduction process preserved the DC‐ssDNA linkage in DNC‐a and DNC‐ac.

In this study, the reduction process employed sodium borohydride as the reducing agent and ethanol as the solvent. Therefore, we hypothesized that the unreacted R—CHO in DNC‐i underwent a heterogeneous redox reaction, producing carboxylate ions (COO^−^), and alcohols (–OH) (i.e., Cannizzaro reaction) (Figure 1b).^[^ ^37^ ^]^ This redox process likely contributed to the observed reduction in XRD diffraction intensity, indicating a relative amorphization of cellulose BBs during the conversion from DNC‐i/‐ic to DNC‐a/‐ac (Figure S10a–c, Supporting Information).^[^ ^38^ ^]^ Interestingly, *D* and CrI values of DNC‐a/‐ac increased relative to their precursors (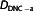 = 3.0 nm, ${\text{CrI}}_{\text{DNC‐a}}$ = 87.0%; 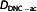 = 3.6 nm, ${\text{CrI}}_{\text{DNC‐ac}}$ = 77.8%) (Table S2, Supporting Information). These increases were accompanied by the formation of networked structure in DNC‐a/‐ac, as shown in TEM images, featuring both rod‐like aggregates ($l_{\text{DNC‐a/‐ac}}$ = 162.9 ± 44.0 nm; $w_{\text{DNC‐a/‐ac}}$ = 13.0 ± 4.7 nm) and amorphous sheets (Figure 3d, S11, Supporting Information). These observations suggested that the large portion of crystalline cellulose lost during reduction process contributed to the overall decrease of diffraction intensity, while the remaining crystalline regions that were larger in size was correlated to the increase in *D* and CrI values. The successful reduction of C=N bond in DNC‐i/‐ic and the presence of ssDNA in DNA‐a/‐ac were further confirmed via UV‐vis analysis. The disappearance of the peak assigned to C=N at ≈350 nm, and a slight shift of the characteristic peak of ssDNA in DNC‐ a and DNC‐ac to 288 and 291 nm, respectively, collectively represented a successful preservation of ssDNA (Figure 3e). We aimed to test the consistent alignment of data collected from the TEM, XRD, and UV‐vis analyses by comparing those obtained from DCda and DCda‐a. Unlike in the cases of DNC‐a/‐ac, the diffraction intensity of DCda‐a was significantly increased compared to that of DCda (Figure S12a, S12b, Supporting Information). While minimal changes were observed from *D* values, a significant increase in the CrI values was observed (${\text{CrI}}_{\text{DCda}}$ = 74.9%; ${\text{CrI}}_{\text{DCda‐a}}$ = 87.1%) (Table S2, Supporting Information). This phenomenon may be attributed to the formation of irregularly shaped nano‐/micro‐aggregates that were revealed in the TEM images of DCda‐a (Figure S12c–e, Supporting Information). The absence of the C=N peak at 359 nm in the UV‐vis spectrum of DCda‐a also confirmed a successive reduction of DCda (Figure S12f, Supporting Information). Collectively, these observations confirmed that that DNC‐a and ‐ac were produced by the reduction of C=N bonds in DNC‐i and ‐ic, respectively.

The DNA‐mediated self‐assembly of DNC‐a and DNC‐ac into slab ‐like microstructures (DNC‐H) was observed in TEM images, showing cellulose aggregates with dimensions of $l_{\text{DNC‐H}}$ = 2.2 ± 0.5 μm and *w*_DNC‐H_ $w_{\text{DNC‐H}}$ = 1.0 ± 0.2 μm (Figure 3a, 1c‐f). Individual rod‐like NPs within DNC‐H measured *w* = 14.4 ± 2.7 nm, with gaps between local aggregates measuring approximately $l_{\text{DNC‐H, Gap}}$ = 141.3 ± 16.1 nm and $w_{\text{DNC‐H, Gap}}$ = 17.6 ± 2.3 nm. These attributes, also confirmed via atomic force microscopy (AFM), indicated that DNC‐H was indeed a hybridized assembly of DNC‐a and DNC‐ac (Figure 3f, S13, Supporting Information). In contrast, when ssDNA sequences were modified to prevent hybridization (seq‐X), no microstructures were observed (DNC‐nH) (Figure S14, Supporting Information). DNA hybridization in DNC‐H may have also induced a slight red‐shift in the ssDNA peak to 295 nm in its UV‐vis spectrum (Figure 3e). We aimed corroborate these observations by the changes in *ζ* values pre‐ and post‐hybridization, and by the assembly of DNC‐a on polystyrene (PS) NPs, functionalized with complementary ssDNA (seq‐C). When ssDNAs, seq‐M ($\zeta_{\text{seq‐M}}$ = −6.2 ± 1.8 mV) and seq‐C ($\zeta_{\text{seq‐C}}$ = −7.6 ± 2.3 mV), alone were mixed to form duplex structures, the *ζ* value decreased ($\zeta_{\text{seq‐M + seq‐C}}$ = −9.2 ± 2.4 mV). This trend was consistently observed from those of DNC‐a, ‐ac, and ‐H ($\zeta_{\text{DNC‐H}}$ = −19.0 ± 2.5 mV) but not from DNC‐nH ($\zeta_{\text{DNC‐nH}}$ − 12.7 ± 1.2 mV) (Table S1, Supporting Information).

To validate DNA hybridization as the driving force for DNC‐H formation, DNC‐a was assembled onto PS NP functionalized with seq‐C (seq‐C@PS). As a control group, DNC‐ac, also containing seq‐C ssDNA, was mixed with seq‐C@PS by the hypothesis that this mixture would not promote DNA hybridization (Figure S15a, Supporting Information). The binding of DNC‐a to seq‐C@PS surface may affect the dispersity of PS core in aqueous solution due to the rich –OH groups of cellulose BBs. Specifically, we hypothesized that the centrifugation of the DNC‐a/seq‐C@PS suspension would yield DNA‐mediated DNC/PS hybrids from the supernatant, and unreacted PS NPs from the precipitates (Figure S15a, Supporting Information). Demonstratively, the supernatants of the centrifuged DNC‐a/seq‐C@PS and DNC‐ac/seq‐C@PS mixtures were collected and dried into pink and white powders, respectively (Figure S15a, Supporting Information). When they were examined under the gel‐fluorescence instrument, the hybridized DNC‐a/seq‐C@PS powders showed a tenfold increase in fluorescence intensity compared to a non‐hybridized control DNC‐ac/seq‐C@PS, presumably due to the larger conjugation between PS NPs and cellulose BBs (Figure S15b, Supporting Information). The TEM analysis confirmed successful binding of DNC‐a to seq‐C@PS surfaces ($l_{\text{DNC‐a/seq‐C@PS}}$ = 154.0 ± 19.3 nm; $w_{\text{DNC‐a/seq‐C@PS}}$ = 11.1 ± 5.3 nm), while no such interaction was observed from DNC‐ac/seq‐C@PS (Figure S16, Supporting Information). These control experiments monitoring *ζ* changes and producing DNA‐mediated DNC/PS hybrids demonstrated the specificity and efficacy of DNA hybridization in driving cellulose BB self‐assembly.

In order to verify that assembly with DNA‐functionalized CNC BBs is indeed based on DNA hybridization, we even explored the effects of introducing self‐complimentary DNA strands (seq‐S) into the production of DNCs.

Self‐complementary ssDNA, by the nature of its base‐pairing, may form hybridized structures in aqueous solutions. However, during the synthesis of seq‐S functionalized DC (DNC‐is, DNC‐as), it was hypothesized that these duplex bonding structures may be disrupted in the THF/CH_3_COOH solvent environment, leading to ssDNA‐ grafting pattern similar to those observed with seq‐M and seq‐C. Demonstratively, the IR spectra of DNC‐is exhibited characteristic peaks of the aromatic C—N group and C=N bond at 1277 cm^−1^ and 1625 cm^−1^, respectively (Figure S17a, S17b, Supporting Information). Similarly, the UV‐vis spectrum of DNC‐is showed blue‐shifted cellulose‐, red‐shifted DNA‐, and imine peaks at 264, 270, and 354 nm, respectively (Figure S17c, S17d, Supporting Information). Morphological analysis of DNC‐is revealed spherical NPs with $d_{\text{DNC‐is}}$ = 96.4 ± 26.2 nm (Figure S18a–c, Supporting Information). On the contrary, the reduction of DNC‐is to DNC‐as ($\zeta_{\text{DNC‐as}}$ = −19.6 ± 1.8 mV) may have led notable changes in cellulose‐DNA chemical structures. While the dimensions of DNC‐as ($l_{\text{DNC‐as}}$ = 186.3 ± 32.7 nm, $w_{\text{DNC‐as}}$ = 14.7 ± 4.7 nm) were similar to those of DNC‐a/‐ac (Figure S18d–f, Supporting Information), the IR spectrum of DNC‐as exhibited a sharp peak at 1637 cm^−1^, corresponding to DNA signals observed in neat DNA^[^ ^39^ ^]^ while the characteristic peaks of carboxylate ions at 1556 and 1409 cm^−1^ were still present (Figure S3, S17a, S17b, Supporting Information). UV‐vis analysis suggested successful reduction of the C=N bond in DNC‐is by the disappearance of the C=N peak at 354 nm, while the DNA peak shifted further to 299 nm, suggesting unique interactions between DNA and the cellulose BB core in DNC‐as (Figure S17c, S17d, Supporting Information). In the case of DNC‐as, microscale slab‐like structure DNC‐sH ($\zeta_{\text{DNC‐sH}}$ = −9.9 ± 3.1 mV) was produced when additional volume of DNC‐as was added into the initial suspension (Figure S18g, Supporting Information). Moreover, the blue‐shift of DNA peak to 296 nm may provide further insight that the self‐assembly of cellulose BBs functionalized with DNAs with self‐complementary hybridization motif might be driven by different interparticle interaction compared to DNC‐a/‐ac (Figure S18g, Supporting Information). These findings provide further proof validating our method of producing self‐assembled cellulose microstructures by DNA hybridization, and demonstrate the feasibility of tailoring DNA‐mediated self‐assembly through hybridization motifs, paving the way for further explorations.

## 3 Conclusion

In summary, this study provides the first demonstration of dynamic morphological transformation and control, and DNA‐mediated self‐assembly of CNC BBs into microscale slab‐like architectures in colloidal states. Our results obtained from FTIR, UV‐vis, and TEM analyses revealed a click‐chemistry mediated dynamic morphological transformation of rod‐like DC into spherical NPs (DNC‐i; $d_{\text{DNC‐i}}$ = 91.1 ± 21.9 nm), followed by their conversion back into rod‐like NPs (DNC‐a; $l_{\text{DNC‐a}}$ = 162.9 ± 44.0 nm; $w_{\text{DNC‐a}}$ = 13.0 ± 4.7 nm) via reduction of imine bonds. DNA hybridization between complementary DNC‐a BBs produced slab‐like cellulose microstructures (DNC‐H; $l_{\text{DNC‐H}}$ = 2.2 ± 0.5 μm; $w_{\text{DNC‐H}}$ = 1.0 ± 0.2 μm). Control experiments with decylamine‐functionalized DC confirmed that imine linkages between DC and amine‐containing moieties may be one of the major driving forces of the morphological transitions. Analyses of XRD patterns, crystallite sizes, and crystallinity indices suggested reduction processes removed amorphous cellulose components, leaving more crystalline cellulose regions that facilitate microstructure self‐assembly via DNA hybridization. The feasibility of this approach was further validated by inducing self‐assembly of DNCs onto DNA‐functionalized PS NPs, successfully yielding DNC/PS hybrids.

Our work establishes a pathway that bridges the strategic gap between self‐assembly methods of cellulose and other anisotropic BBs, while simultaneously addressing the challenges in DNA nanotechnology by providing a platform for the development cost‐efficient well‐defined organic architectures with programmable geometry and length scales. The resulting bioderived BBs which exhibit intrinsic anisotropy, simple chemical modifications, and biocompatibility hold promise for applications in medicine, energy, and soft robotics, particularly as carriers or scaffolds. Future research could explore various experimental parameters such as the types of cellulose BB cores, ssDNA length, and hybridization motifs to refine self‐assembly structures and expand the library of polysaccharidal BB architectures. This expanded understanding of cellulose–DNA interactions could unlock unexplored materials designs and properties.

## Conflict of Interest

The authors declare no conflict of interest.

## Supporting information

Supplementary Material

## Data Availability

The data that support the findings of this study are available in the supplementary material of this article.
