# Supplementary material for: Dynamic Morphological Transformation and Self‐Assembly of DNA‐Functionalized Cellulose Nanocrystal Building Blocks
Source: ChemSusChem. 2025 Jun 17;18(15):e202500341. doi: 10.1002/cssc.202500341 (PMC12302319; doi:10.1002/cssc.202500341)
Supplement: Supplementary file 1 — Supplementary Material [file CSSC-18-e202500341-s001.pdf]

Supplementary Information

Dynamic Morphological Transformation and Self-Assembly of DNA-functionalized Cellulose Nanocrystal Building Blocks

Jinsu Park[a], Youngeun Kim*[a],[b], Seung-Yeop Kwak*[a],[b],[c]

[a] J. Park, Dr. Y. Kim, Dr. S.-Y. Kwak
Department of Materials Science and Engineering, Seoul National University, 1 Gwanak-ro, Gwanak-gu, Seoul, 08826, South Korea;
E-mail: youngeunkim@snu.ac.kr; sykwak@snu.ac.kr

[b] Dr. Y. Kim, Dr. S.-Y. Kwak
Research Institute of Advanced Materials (RIAM), Seoul National University, 1 Gwanak-ro, Gwanak-gu, Seoul, 08826, South Korea

[c] Dr. S.-Y. Kwak

Institute of Engineering Research, Seoul National University, 1 Gwanak-ro, Gwanak-gu, Seoul, 08826, South Korea

Materials and Methods

Materials Cellulose nanocrystal (CNC) was purchased from Nanografi Nano Technology and was used as received. Sodium periodate was purchased from Junsei Chemical Co., Ltd. and was used as received. Tetrahydrofuran and ethanol were purchased from Dae Jung Chemicals, Inc. 2-methylpyridine borane (picoline borane), acetic acid, latex beads (carboxylate-modified polystyrene, fluorescent red), 2-(N-morpholino)ethanesulfonic acid (MES) hydrate, ethanolamine, N-(3-Dimethylaminopropyl)-N′-ethylcarbodiimide hydrochloride (EDC) were purchased from Sigma-Aldrich. Sodium borohydride was purchased from TCI Sejin CI. Oligonucleotide strands were purchased from Integrated DNA Technologies, inc. and were used as received. The purchased oligonucleotides (dubbed seq-M, seq-C, seq-S, seq-X) are:

seq-M 5' {AmMC6} TTT TTT TTT TTT TCA GGA TGC ATG CTA GTC GC 3'

seq-C 5' {AmMC6} TTT TTT TTT TTT TTT TTT TTG CGA CTA GCA TGC ATC CTG A 3'

seq-S 5' {AmMC6}TTT TTT TTT TTT TTT TTT TTT CAG GAT GCA TGC TAG TCG C 3'

seq-X 5' {AmMC6} TTT TTT TTT TTT TCT GGT AGG ATG GTA GTC GC 3'

Synthesis of DC. Dialdehyde cellulose was synthesized by following previously reported procedure.[50] Briefly, a 3 g of CNCs were dispersed in DI water (2.4 wt%). In a separate vial, a sodium periodate ($NaIO_4$) solution (1.2 molar excess to CNC) was prepared (7.87 wt%). After thoroughly stirring the CNC dispersion, the $NaIO_4$ solution was added dropwise in a N_2-purged environment. The reaction was held for approximately 2.5 hrs. Once the reaction was at halt, the obtained mixture was washed with DI water three times, and freeze dried. The resulting product was then freeze-milled into white powders, and stored at below -10 ℃.

Synthesis of imine-linked DC-ssDNA/-decylamine hybrids (DNC-i, DNC-ic, DNC-is, DCda). A solvent mixture containing 25 g of tetrahydrofuran and 1 mL of acetic acid (pH = 4 – 6) was prepared (THF/CH_3COOH). Then approximately 50 mg of DC was dispersed in the THF/CH_3COOH to result 0.4 wt% suspension. After sonicating the suspension using probe ultrasonicator, at room temperature, 100 uL of 79.34 μM of ssDNA aqueous solutions (seq-M, seq-C, seq-S, seq-X) and 100 mM picoline borane/THF solutions were added dropwise. After leaving the mixture stirred for 15 min, the mixture was transferred to a 60 ℃ oil bath and further stirred for 24 hrs. In about 20 minutes, the reactant mixture started changing its color to yellow. For the synthesis of DCda, the ssDNA solution was replaced with a decylamine/THF solution (79.34 μM). The synthesis of DCda took approximately 4 days. After 24 hrs, approximately 200 mg of picoline borane powders were directly added into the reaction mixture and further stirred for 6 hrs at 50 ℃. Once the reaction was put to halt, the obtained products were washed with the solvent three times and freeze-dried to yield yellow powders. These powders were stored at below -10 ℃.

Reduction of DNC-i, DNC-ic, DNC-is, and DCda. All imine-linked DC-ssDNA/-decylamine hybrids were dispersed in ethanol (0.25 wt%). Then, in an open vial, excess amounts of sodium borohydrides were added. The reduction process must be held in an open vial as H_2 gas are produced as a byproduct. Once the reaction was at halt, the obtained mixtures were quenched using acetic acid in an ice bath. Approximately 1.5 mL of acetic acid was used to quench 10 mL of the reducing mixture. Upon completing the quenching process, the obtained mixtures were washed with ethanol and DI water three times, and freeze dried. The resulting white powders were stored at below -10 ℃ (DNC-a, DNC-ac, DNC-as, DCda-a).

DNA-mediated hybridization of DNC-a, DNC-ac, and DNC-is (Synthesis of DNC-H, DNC-sH, and DNC-nH). 0.1 wt% aqueous dispersions of DNC-a, DNC-ac, and DNC-is were sonicated using a bath-sonicator. Then 1 mL of each dispersion were drawn and transferred into separate vials. For the production of DNC-H, 200 uL of DI water, and 400 uL of DNC-a and DNC-ac solutions were mixed together and stirred. In the case of DNC-sH, 200 uL of DI water and 800 uL of DNC-as were stirred together. DNC-nH was prepared by mixing DNC-a with seq-X functionalized DNCs.

Preparation of DNC/PS hybrids. ssDNA (seq-C)-functionalized PS NPs were prepared following widely available protocol. 20 uL of latex beads were aliquoted in a 2 mL Eppendorf tube, to which 380 uL MES buffer (pH = 4.5) was added. The mixture was centrifuged at 14,000 RPM for 15 min, and the supernatant was separated from the latex solution. Then, 150 uL of 0.1 M MES buffer, 100 uL of 79.34 uM ssDNA (seq-C), and 100 uL of EDC solution (12.5 mg EDC in 250 uL 0.1 MES) were added into this vial. After keeping the sample away from light for more than 2 hrs, 6 uL of 1 M ethanolamine was added into the solution, prior to further centrifugation at 14,000 RPM for 15 min. Once removing the supernatant, the resulting seq-C@PS NPs were stored at below -10 ℃. These seq-C@PS NPs were then redispersed in 1 mL of DI water. After adding 400 uL of DNC-a or DNC-ac solutions, the resulting DNC/PS mixtures were vortexed for 1 min and centrifuged at 15,000 RPM for 35 min. Lastly, supernatants from both DNC-a/seq-C@PS and DNC-ac/seq-C@PS mixtures were aliquoted in a separate 2 mL Eppendorf tube and stored at below -10 ℃.

Materials Characterizations

The ATR-FTIR spectra of the specimens were obtained in their powdered form using the Thermo Scientific Nicolet iS5 instrument and OMNIC software. For UV-visible spectroscopy (Agilent Cary 3500; Cary UV Workstation software) and zeta potential measurements (Anton Paar Litesizer 500; Kalliope software), all imine-linked DC-ssDNA/-decylamine (DNC-int, DNC-i, DNC-ic, DNC-is, DNC-iX, DCda) were dispersed in 8:2 (v/v) acetonitrile/dimethyl sulfoxide (0.12 wt%), while those that did not contain imine bonds (DC, DNC-a, DNC-ac, DNC-as, DNC-aX, DCda-a, DNC-H, DNC-sH, DNC-nH, seq-M, seq-C, seq-C, seq-X, seq-C@PS) were dispersed in DI water (0.1 wt%). Approximately 15 mg of all specimens were used in their powdered forms to obtained XRD patterns (Bruker D8 Advance). The powder XPS analyses of imine-linked DC-ssDNA/-decylamine samples were performed using the Thermo Fisher Scientific NEXSA instrument. The surface topology and length profile of DNC-H was obtained by atomic force microscopy (AFM) analysis (Park Systems NX-10). The TEM images presented in this study were obtained using the JEM-2100PLUS instrument (200 kV). The fluorescence image of DNC-a/seq-C@PS and DNC-ac/seq-C@PS hybrids were obtained using the Azure 600 instrument.

Table 1. Measured zeta potential values of the DC-DNA hybrids presented in this study.

	Zeta-Potential (ζ) (mV)
Sequence M (seq-M)	-6.2 ± 1.8
Sequence C (seq-C)	-7.6 ± 2.3
seq-M + seq-C	-9.2 ± 2.4
DC	-27.3 ± 2.6
DNC-int	-32.0 ± 1.2
DNC-i (seq-M)	-44.6 ± 1.7
DNC-ic (seq-C)	-43.8 ± 0.9
DNC-a (seq-M)	-17.7 ± 1.5
DNC-ac (seq-C)	-17.6 ± 1.9
DNC-H	-19.0 ± 2.5
DNC-nH	-12.7 ± 1.2
DCda	-31.1 ± 3.1
DCda-a	-6.2 ± 1.0

Table 2. Crystallite sizes (D) and crystallinity indices (CrI) of CNC, DC, DNC-int, -i, and -ic were calculated using the Scherrer equation: (*D*=(Kλ)/βcos(θ)), and the Segal method: ($[\frac{I_{200}-I_{amorphous}}{I_{200}}] \times 100$ %), in which K = 0.89; λ = 1.5406 Å; β = full width at half max values at 2θ= 22.3°; and Iamorphous, I200 were the peak intensity values of the peaks at 2θ= 19.5° and 22.3°, respectively.

	D (nm)	CrI (%)
CNC	3.8	87.0
DC	2.7	77.8
DNC-int	2.7	61.9
DNC-i	2.6	77.0
DNC-ic	2.6	66.4
DNC-a	3.0	87.0
DNC-ac	3.6	77.8
DCda	3.0	74.9
DCda-a	3.0	87.1

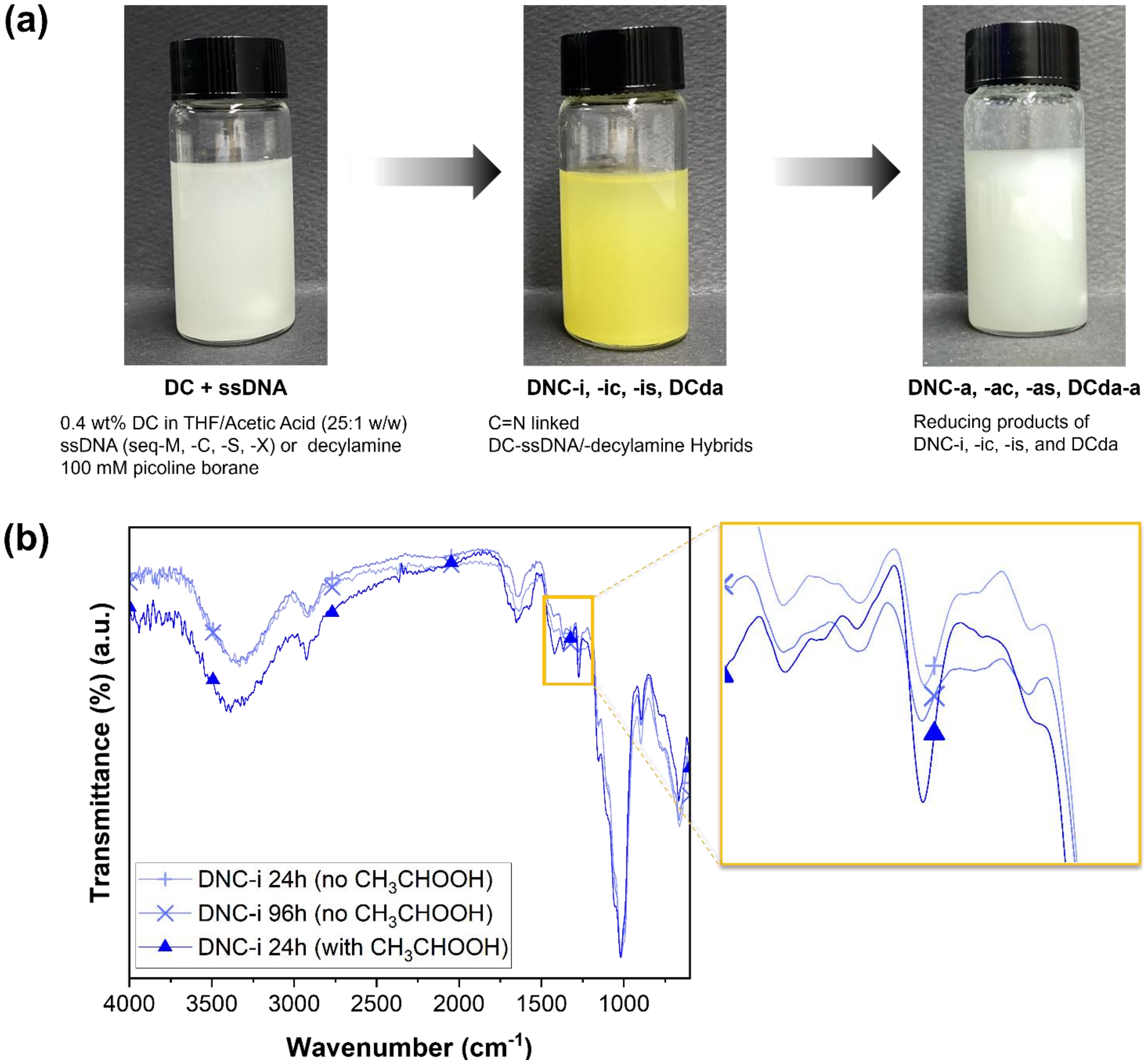

Figure S1. (a) A schematic showing the visible changes observed from the reaction products obtained at each synthesis steps Notably, the C=N linked products exhibited yellow color. (b) IR spectra of the specimens obtained from day 1 and 4 without using acetic acid showed lower signal intensity of ssDNA at 1277 cm^{-1}, as compared to that observed from conventional DNC-i. The DC–ssDNA reaction in neat THF required prolonged reaction times (1– 4 days). Interestingly, a gradual decrease in the pH level of the DC–ssDNA/THF mixture was observed, from approximately 7–8 to 4–6 over time, presumably due to a slow proton transfer process. To accelerate this reaction, a THF/acetic acid (CH_3CHOOH) (pH ~ 4–6) mixture was used as the solvent. This approach significantly reduced the reaction time to 24 hours and potentially enhanced the efficiency of DC–ssDNA functionalization, as evidence by the IR spectra.

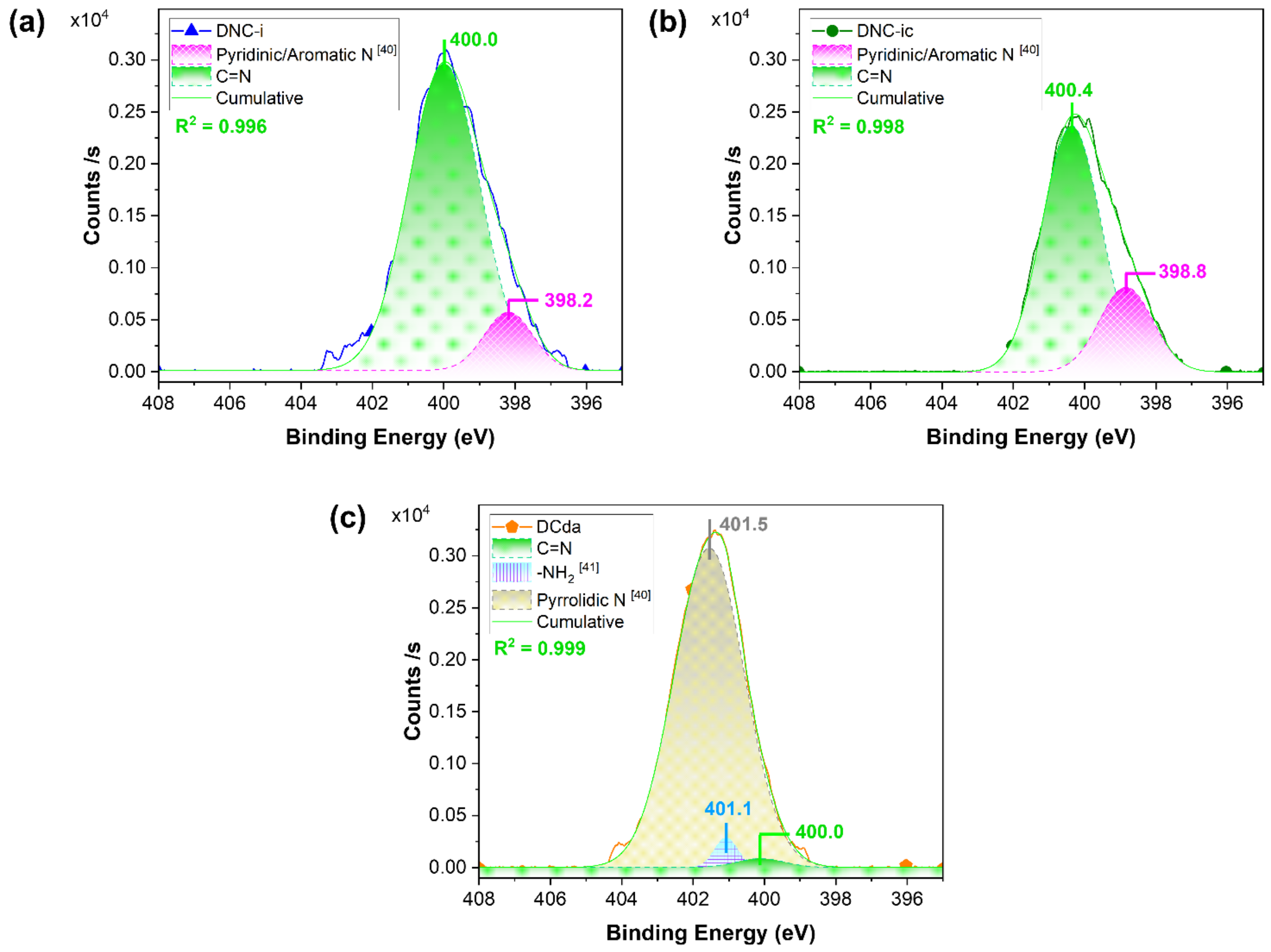

Figure S2. N1s XPS profiles of (a) DNC-i, (b) DNC-ic, and (c) DCda exhibited C=N signals at approximately 400 eV. Especially for the specimens containing ssDNA, pyridinic C-N signals were observed at around 398 eV (DNC-i and DNC-ic).

References

[40] A. Alabadi, H. A. Abbood, Q. Li, N. Jing, B. Tan, *Sci Rep* **2016**, *6*, 38614.

[41] H. Wang, X. Li, Q. Ruan, J. Tang, *Nanoscale* **2020**, 12, 12329.

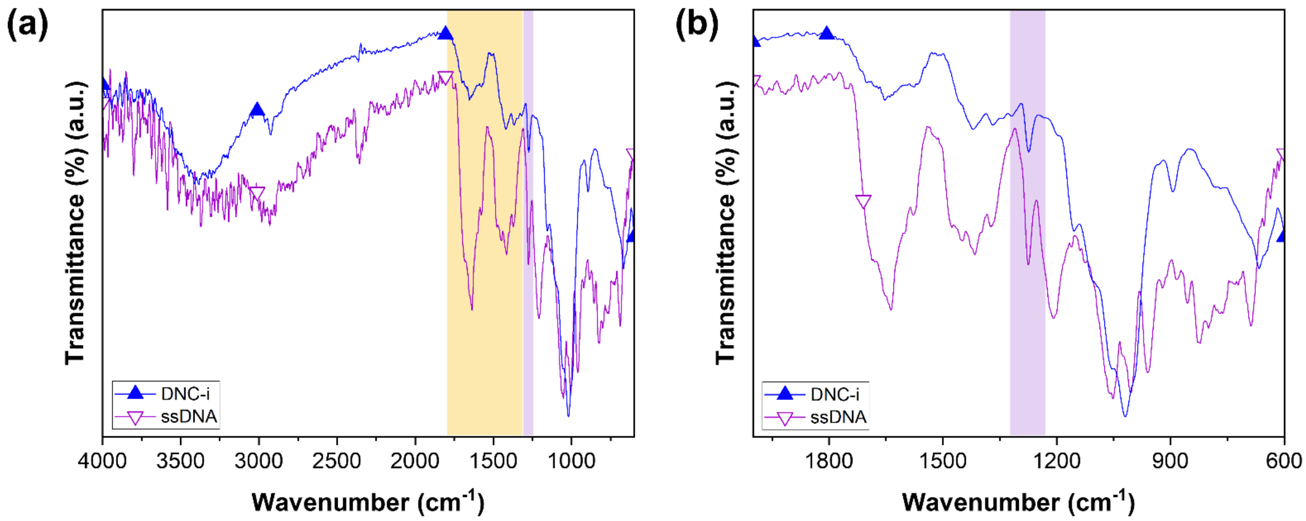

Figure S3. (a – b) The FTIR spectra of DNC-i and neat ssDNA exhibited a sharp signal corresponding to aromatic C-N at 1277 cm^{-1}.

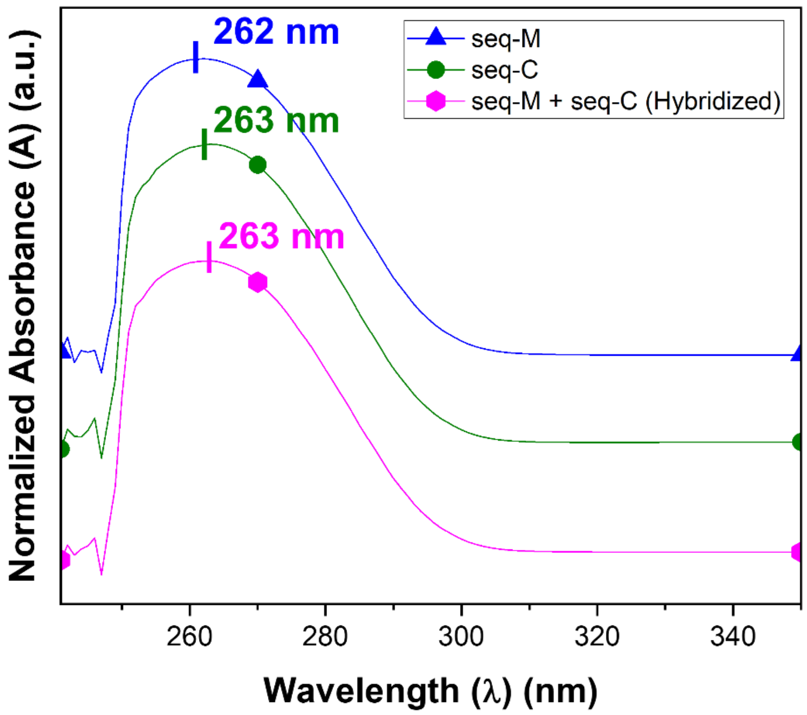

Figure S4. UV-vis spectra of neat ssDNA exhibiting characteristic peaks at approximately 263 nm before and after DNA hybridization.

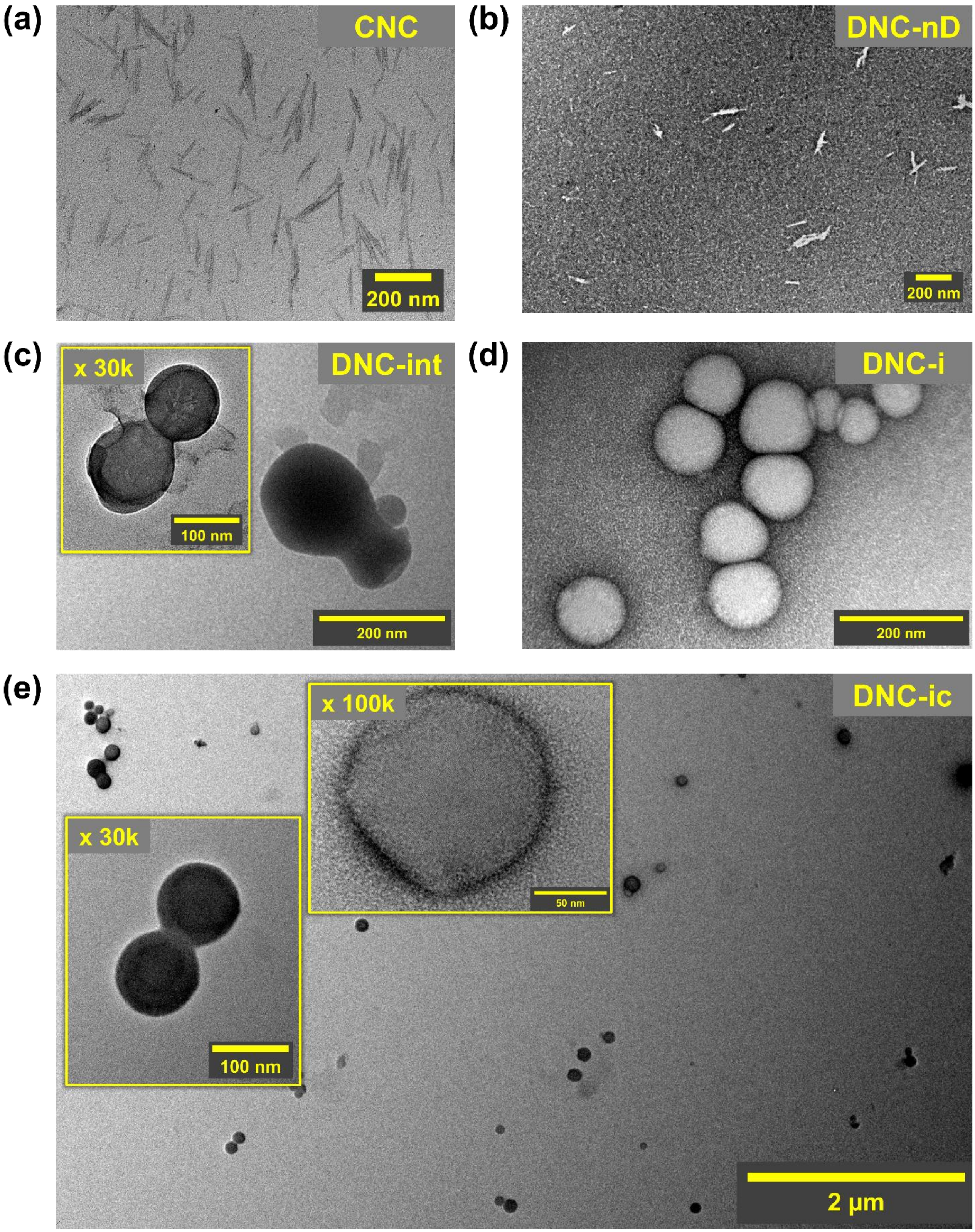

Figure S5. TEM images of (a) neat CNC, (b) DNC-nD that contains no ssDNA, (c) DNC-int, (d) DNC-i, and (e) DNC-ic showing the dynamic morphological transformation of CNCs into spherical DNC NPs upon formation of C=N linkage between DCs and ssDNA.

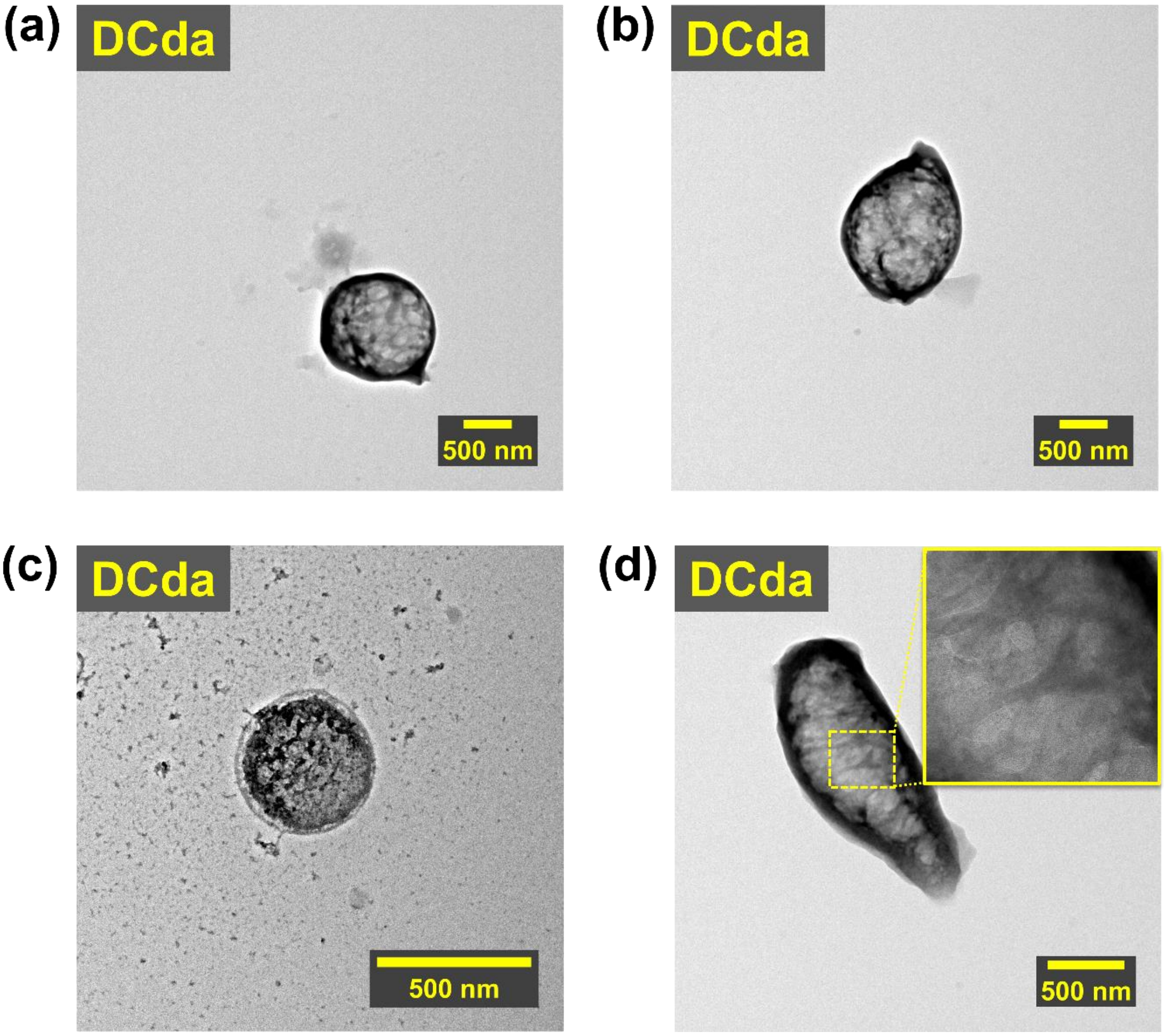

Figure S6. TEM images of (a – d) DCda exhibiting different morphological characteristics.

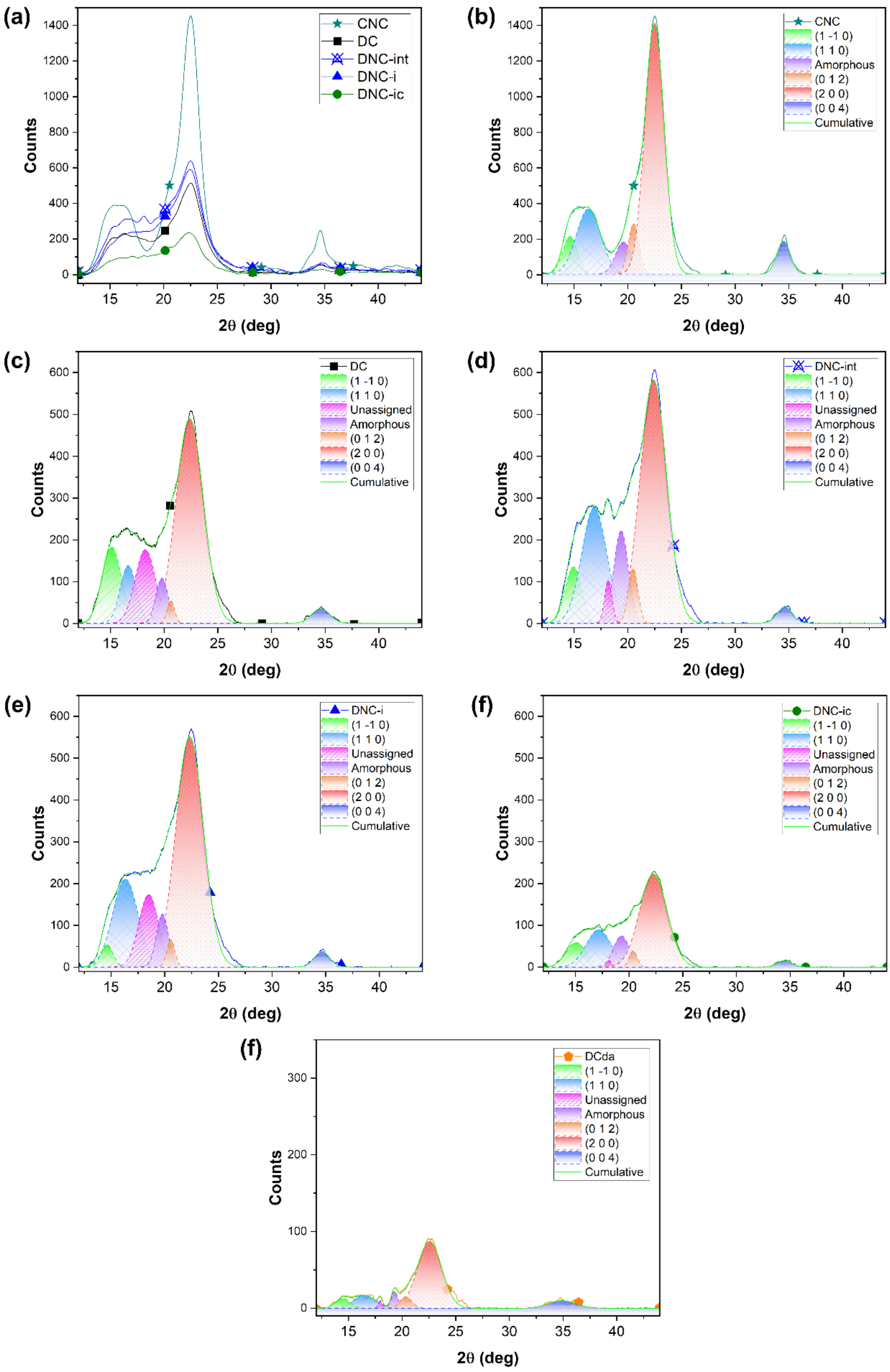

Figure S7. (a) The XRD patterns of CNC, DC, DNC-int, DNC-i, and DNC-ic showed that the more significant reduction in crystalline structure of cellulose BBs was done during the transition from CNCs to DC. The Gaussian-deconvoluted XRD patterns of (b) CNC, (c) DC, (d) DNC-int, (e) DNC-i, (f) DNC-ic exhibited a newly formed peak at 2θ = ~18.3°. The XRD pattern of (f) DCda exhibited significantly reduced crystalline structure which made it challenging to discern the amorphous peak at ~19.2°.

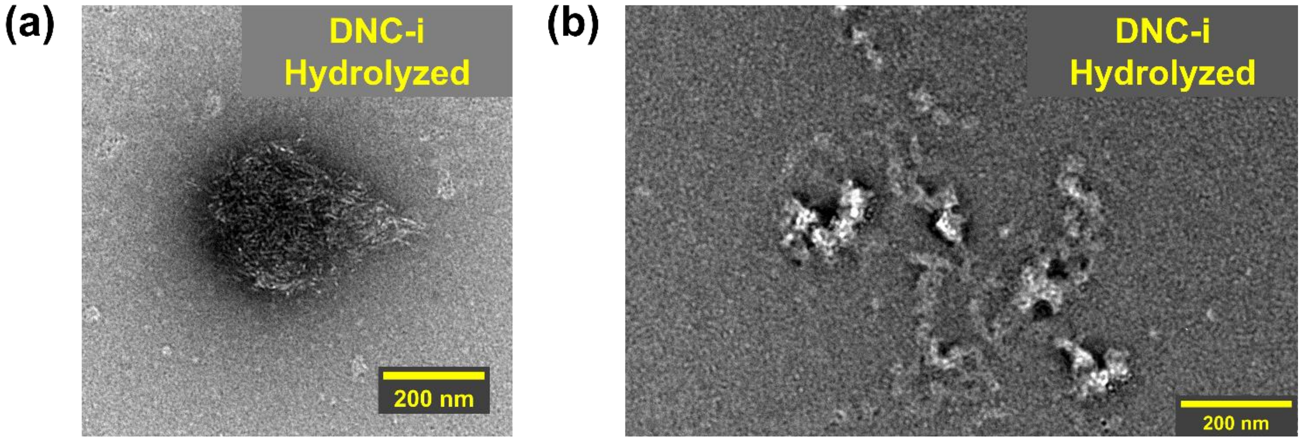

Figure S8. TEM images showing the hydrolysis of DNC-i upon exposure to aqueous environment that reverted it back to (a) DC aggregates or (b) rod-like DC NPs.

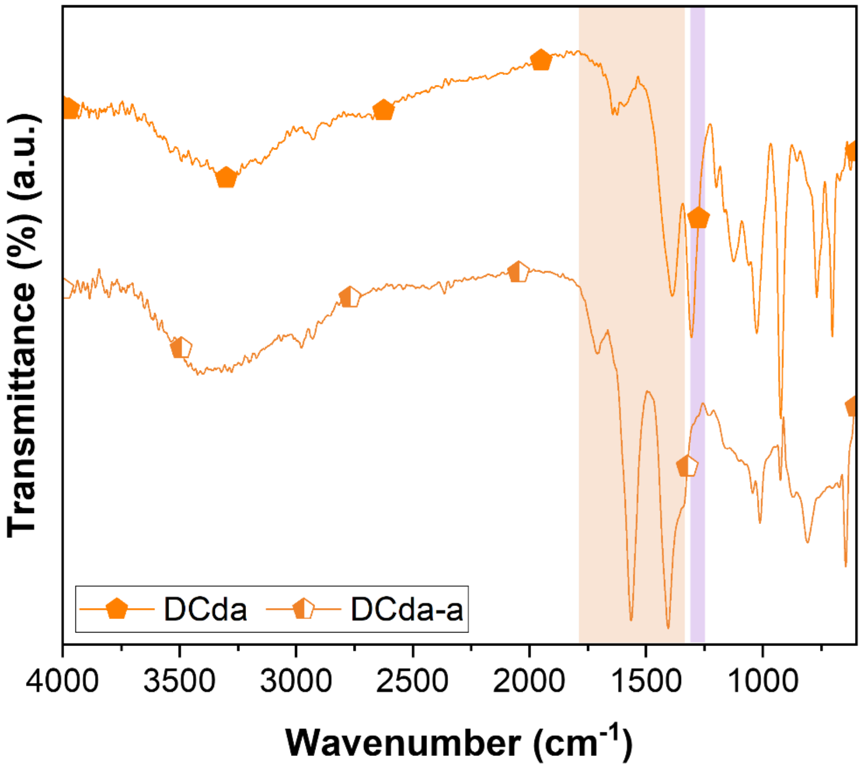

Figure S9. ATR-FTIR spectra of DCDa and DCda-a exhibiting the absence of aromatic C-N peak at 1277 cm^{-1} and the emergence of asymmetric and symmetric carboxylate ion peaks in the 1562 – 1409 cm^{-1} region.

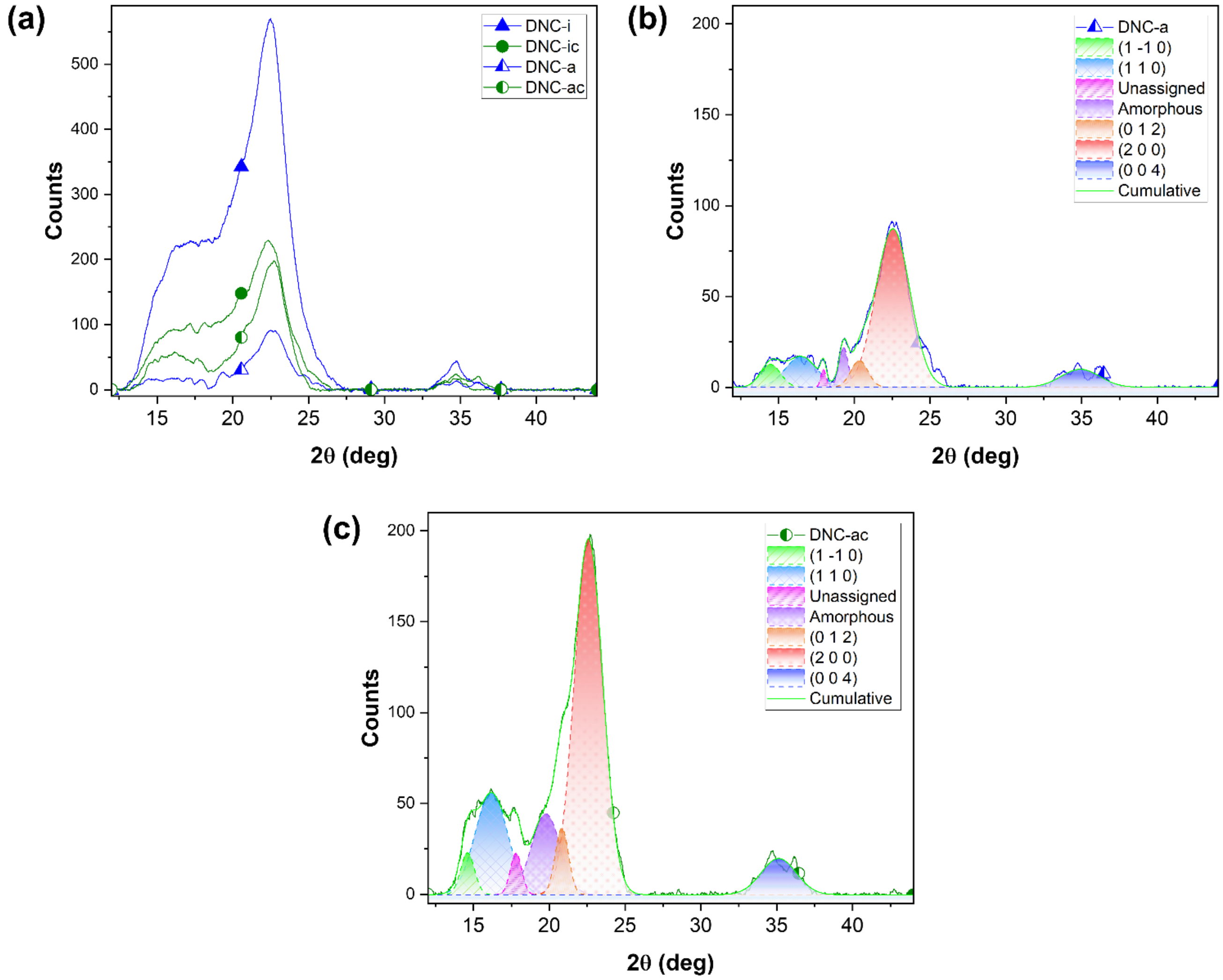

Figure S10. The (a) XRD spectra of DNC-i and DNC-ic, and their respective reducing products (b) DNC-a and (c)DNC-ac exhibited reduction of diffraction intensity of the DNC building blocks after the reducing process.

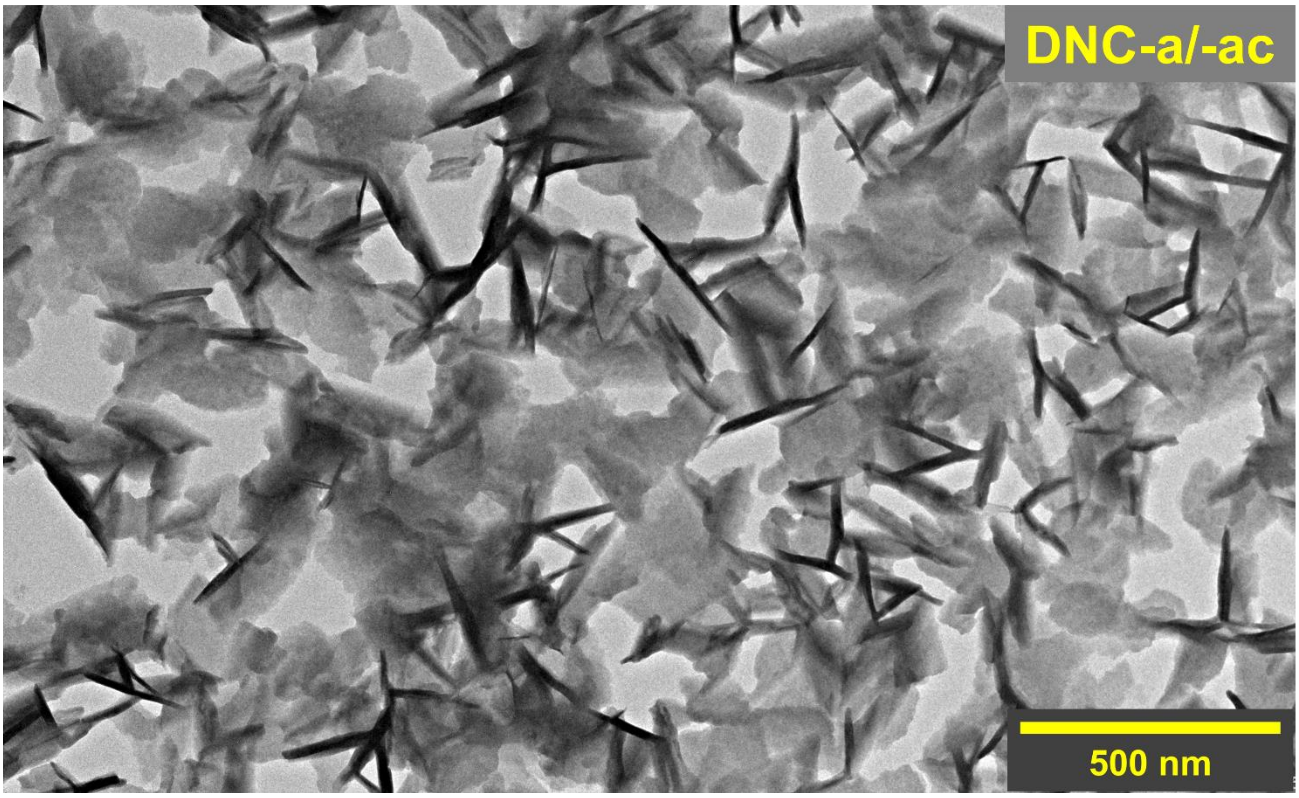

Figure S11. The TEM image of DNC-a/-ac revealed the presence of both rod-like aggregates with dimension of $l_{DNC\text{-}a/\text{-}ac}$ = 162.9 ± 44.0 nm and $w_{DNC\text{-}a/\text{-}ac}$ = 13.0 ± 4.7 nm, and amorphous sheets.

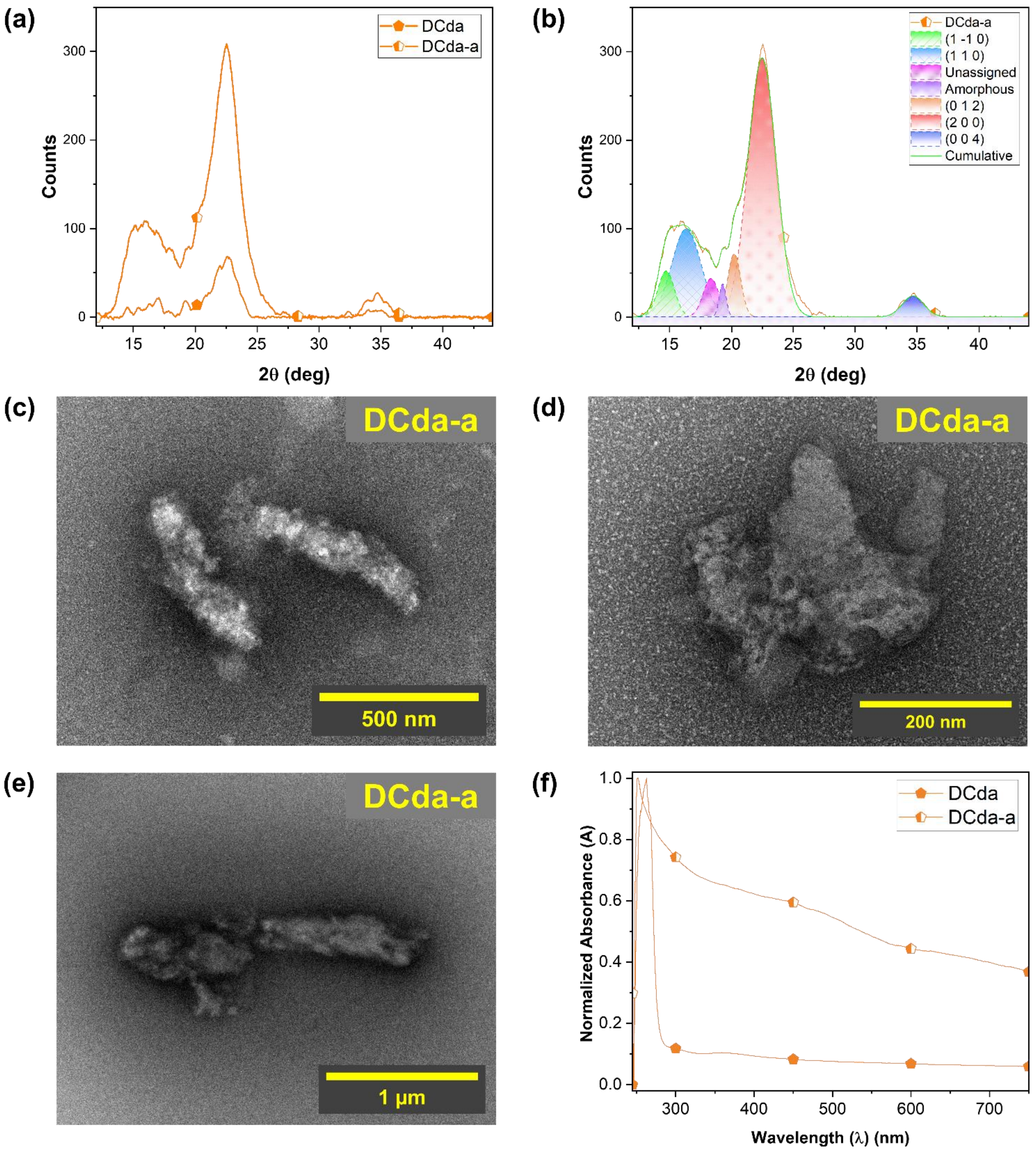

Figure S12. (a) The XRD patterns of DCda and (b) DCda-a also exhibited an increase of diffraction intensity upon reducing the imine-linked DCda. (c – e) The TEM images of DCda-a showed aggregated microstructures. (f) A successful reduction of DCda to DCda-a was observed from the disappearance of the peak at 359 nm in their UV-vis spectra.

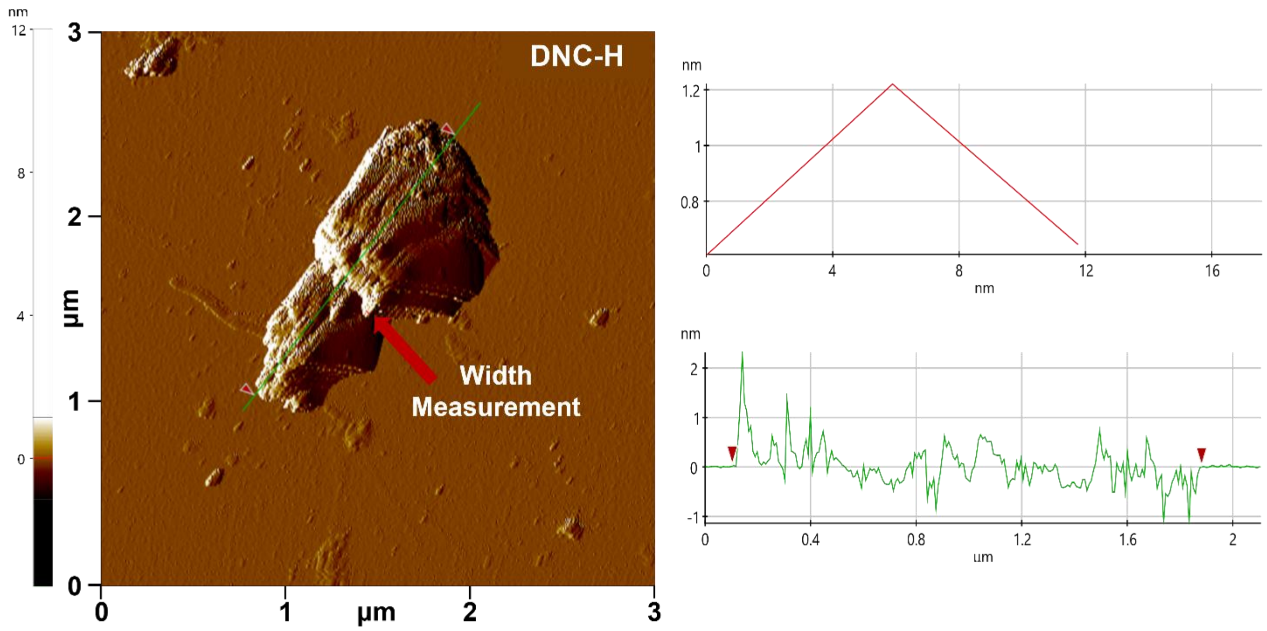

Figure S13. Slab-like cellulose microstructure DNC-H was also confirmed via AFM analysis.

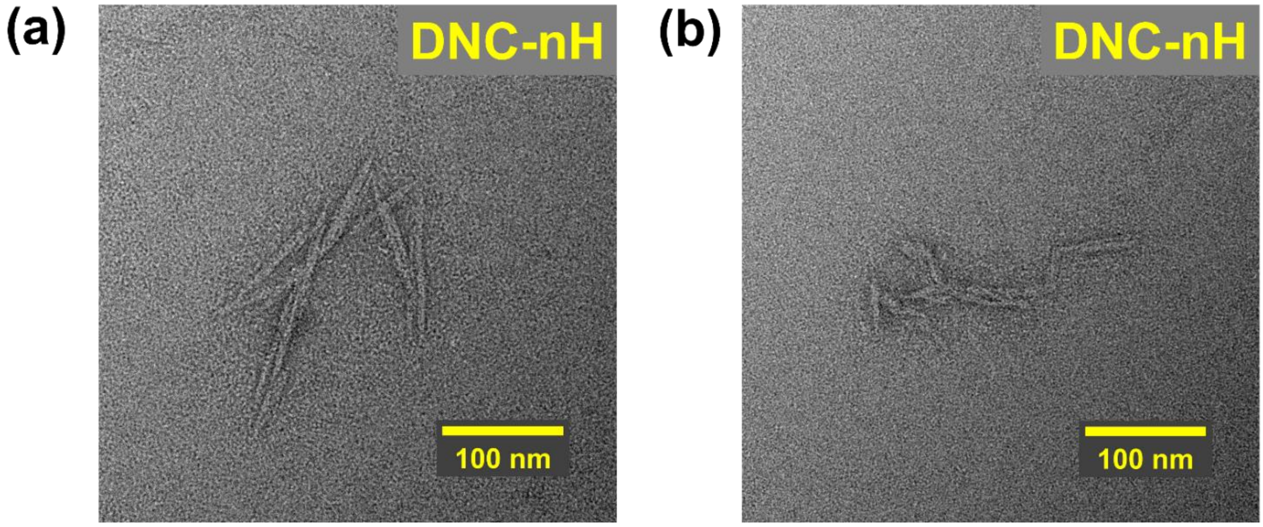

Figure S14. The TEM images of (a – b) DNC-nH exhibited absence of slab-like microstructures without DNA-hybridization.

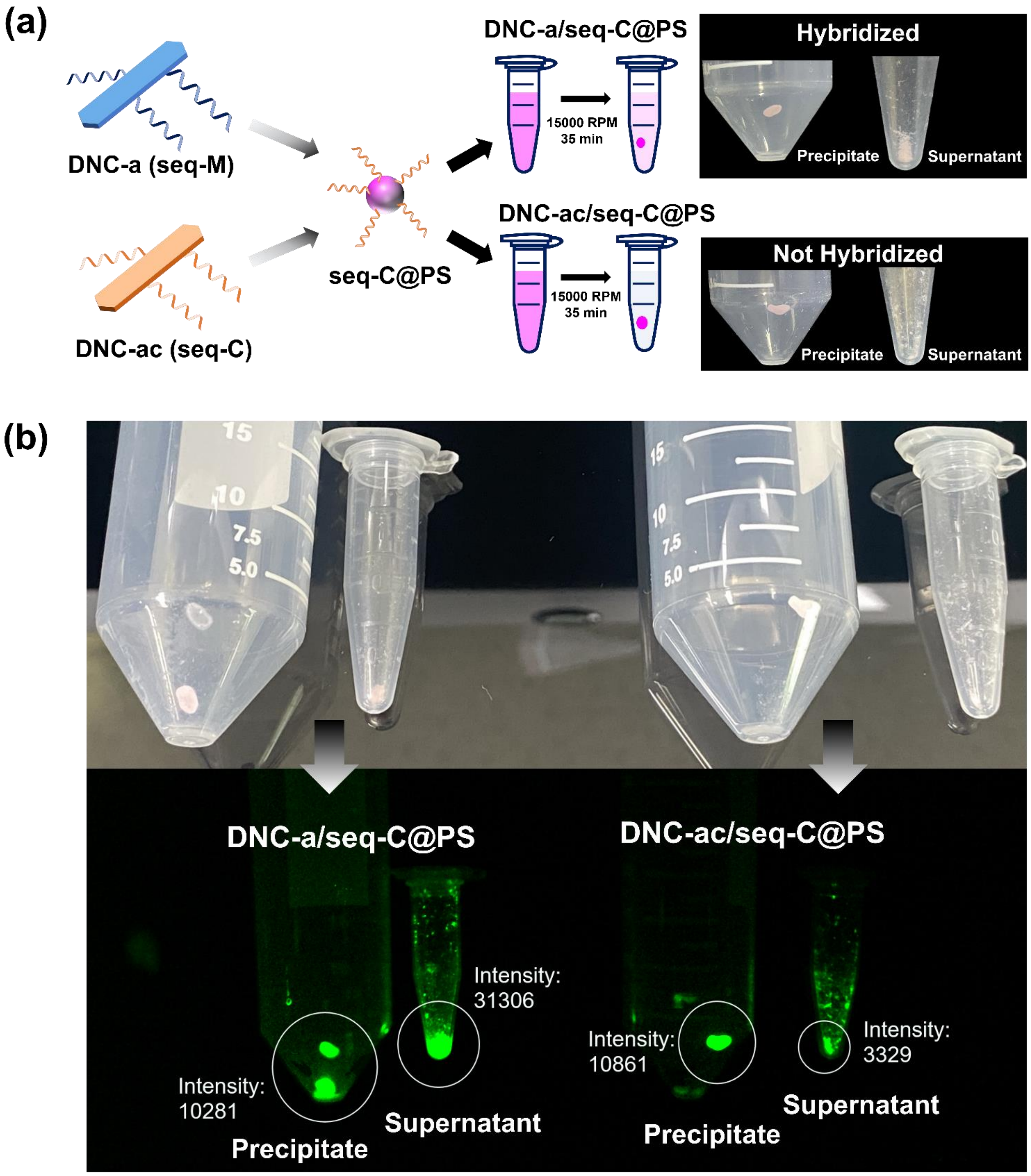

Figure S15. (a) Latex PS NPs were functionalized with seq-C ssDNA. Then, by mixing them with DNC-a (seq-M) and DNC-ac (seq-C), we aimed to obsere the formation of DNC/PS hybrids via DNA hybridization, and test the validity of the construction of DNC-H. After freeze-drying the supernatant of each mixtures: DNC-a/sec-C@PS, DNC-ac/seq@PS, pink powders were only obtained from the former specimen. (b) Stronger fluorescence intensity was observed from the powder obtained from supernatant of DNC-a/sec-C@PS mixture, potentially suggesting a formation of DNC/PS hybrids.

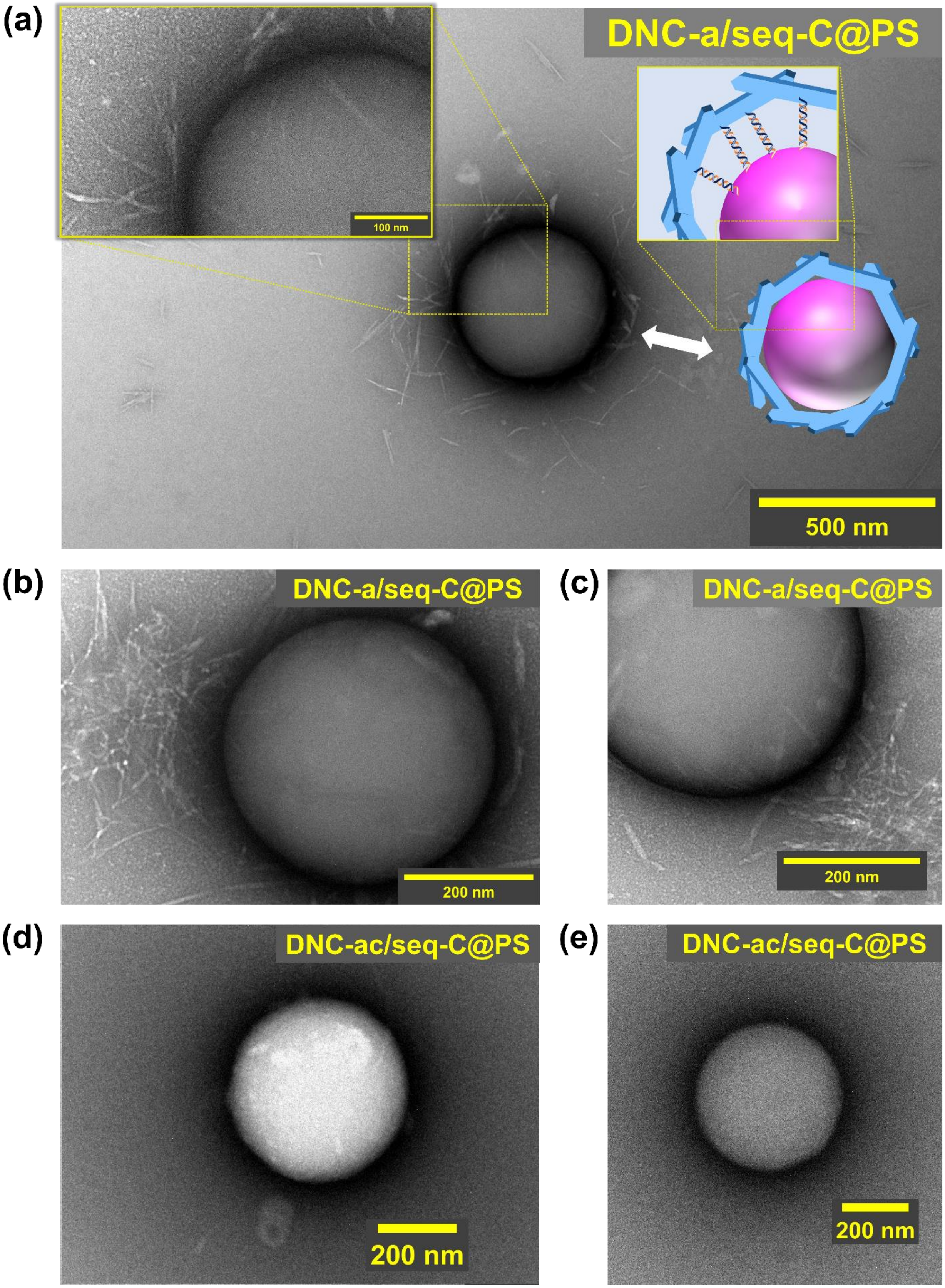

Figure S16. The TEM images of (a – c) DNC-a/seq-C@PS and (d, e) DNC-ac/seq-C@PS showed that the hybridization between seq-M and seq-C between DNC-a and PS NPs afforded the formation of DNC/PS hybrids, thereby validating the construction of DNC-H.

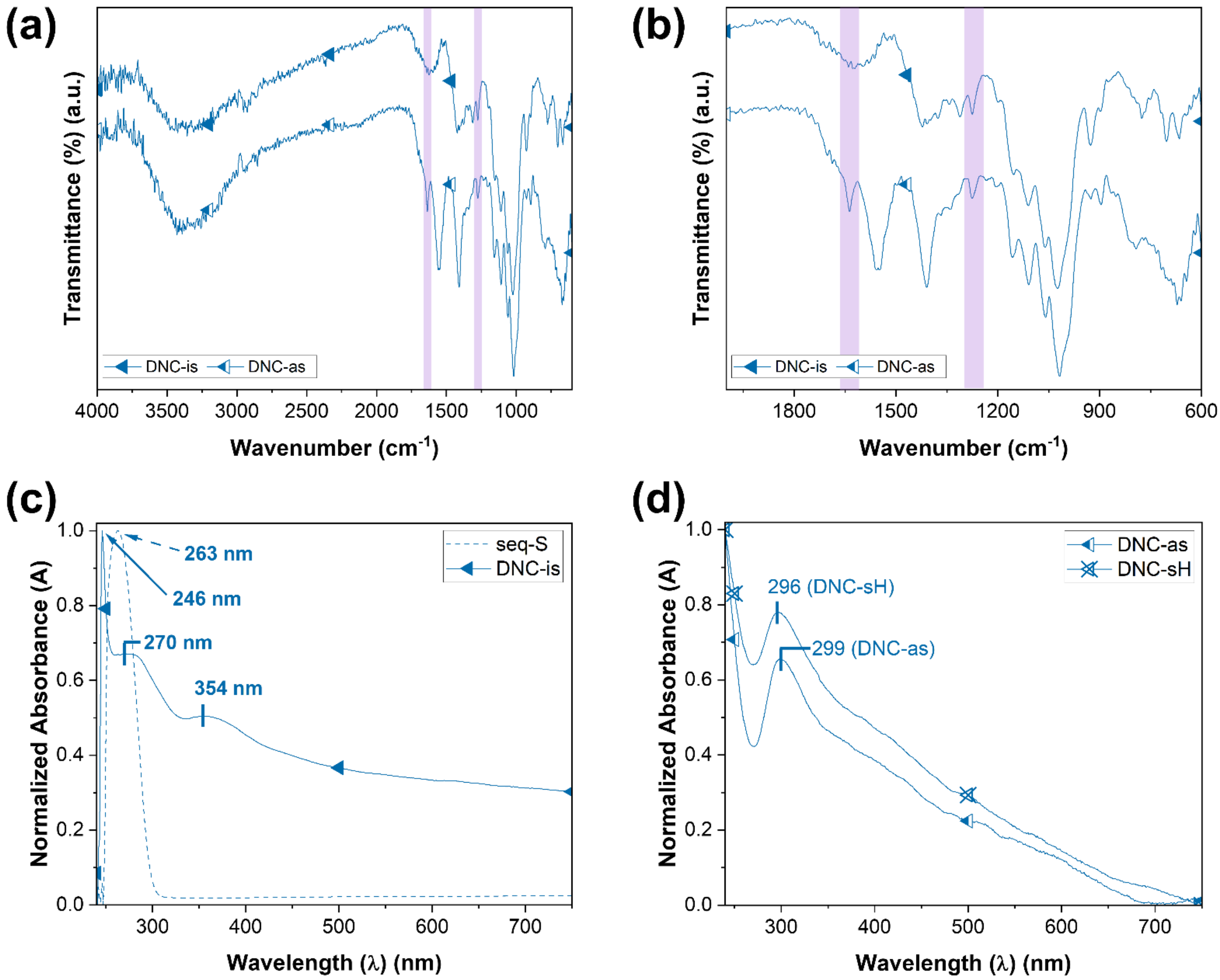

Figure S17. (a, b) The ATR-FTIR and (c, d) UV-vis spectra of DNC-is, -as- and -sH exhibited preservation of aromatic C-N peak at 1277 cm^{-1} and red-shift of ssDNA peak, as also observed from DNC-i, -a, and -H.

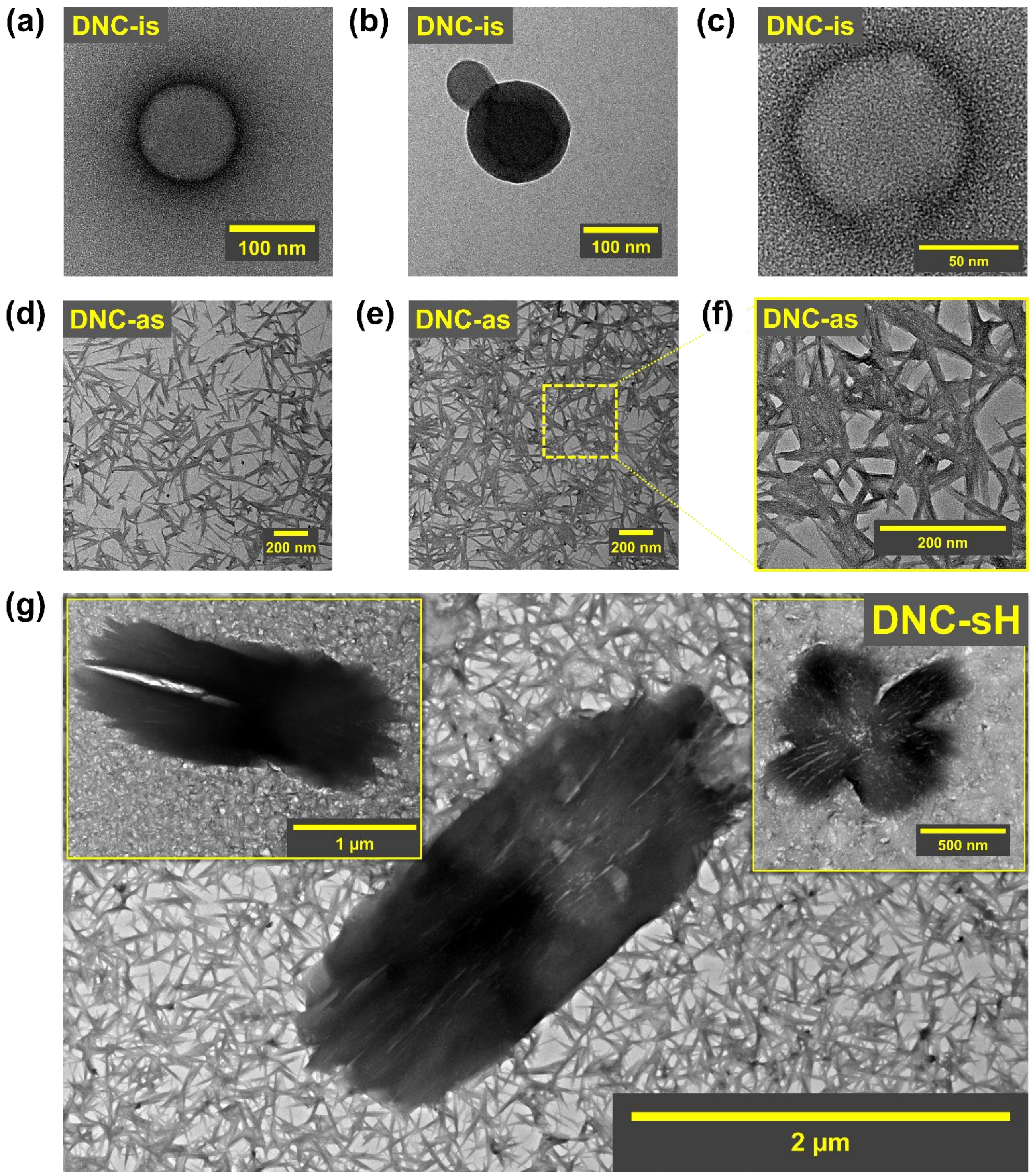

Figure S18. The TEM images of (a – c) DNC-is, (d – f) DNC-as, and (g) DNC-sH also exhibited the similar morphological transformation of cellulose BBs that was observed throughout this study.
